# An integrated genomic approach identifies follistatin as a target of the p63-epidermal growth factor receptor oncogenic network in head and neck squamous cell carcinoma

**DOI:** 10.1093/narcan/zcad038

**Published:** 2023-07-24

**Authors:** Akinsola Oyelakin, Jennifer Sosa, Kasturi Bala Nayak, Alexandra Glathar, Christian Gluck, Isha Sethi, Maria Tsompana, Norma Nowak, Michael Buck, Rose-Anne Romano, Satrajit Sinha

**Affiliations:** Department of Oral Biology, School of Dental Medicine, State University of New York at Buffalo, Buffalo, NY, USA; Department of Biochemistry, Jacobs School of Medicine and Biomedical Sciences, State University of New York at Buffalo, Buffalo, NY, USA; Department of Biochemistry, Jacobs School of Medicine and Biomedical Sciences, State University of New York at Buffalo, Buffalo, NY, USA; Department of Biochemistry, Jacobs School of Medicine and Biomedical Sciences, State University of New York at Buffalo, Buffalo, NY, USA; Department of Biochemistry, Jacobs School of Medicine and Biomedical Sciences, State University of New York at Buffalo, Buffalo, NY, USA; Department of Biochemistry, Jacobs School of Medicine and Biomedical Sciences, State University of New York at Buffalo, Buffalo, NY, USA; Department of Biochemistry, Jacobs School of Medicine and Biomedical Sciences, State University of New York at Buffalo, Buffalo, NY, USA; Department of Biochemistry, Jacobs School of Medicine and Biomedical Sciences, State University of New York at Buffalo, Buffalo, NY, USA; Department of Biochemistry, Jacobs School of Medicine and Biomedical Sciences, State University of New York at Buffalo, Buffalo, NY, USA; Department of Biochemistry, Jacobs School of Medicine and Biomedical Sciences, State University of New York at Buffalo, Buffalo, NY, USA; Department of Biomedical Informatics, Jacobs School of Medicine and Biomedical Sciences, State University of New York at Buffalo, Buffalo, NY, USA; Department of Oral Biology, School of Dental Medicine, State University of New York at Buffalo, Buffalo, NY, USA; Department of Biochemistry, Jacobs School of Medicine and Biomedical Sciences, State University of New York at Buffalo, Buffalo, NY, USA

## Abstract

Although numerous putative oncogenes have been associated with the etiology of head and neck squamous cell carcinoma (HNSCC), the mechanisms by which these oncogenes and their downstream targets mediate tumor progression have not been fully elucidated. We performed an integrative analysis to identify a crucial set of targets of the oncogenic transcription factor p63 that are common across multiple transcriptomic datasets obtained from HNSCC patients, and representative cell line models. Notably, our analysis revealed *FST* which encodes follistatin, a secreted glycoprotein that inhibits the transforming growth factor TGFβ/activin signaling pathways, to be a direct transcriptional target of p63. In addition, we found that FST expression is also driven by epidermal growth factor receptor EGFR signaling, thus mediating a functional link between the TGF-β and EGFR pathways. We show through loss- and gain-of-function studies that FST predominantly imparts a tumor-growth and migratory phenotype in HNSCC cells. Furthermore, analysis of single-cell RNA sequencing data from HNSCC patients unveiled cancer cells as the dominant source of FST within the tumor microenvironment and exposed a correlation between the expression of *FST* and its regulators with immune infiltrates. We propose FST as a prognostic biomarker for patient survival and a compelling candidate mediating the broad effects of p63 on the tumor and its associated microenvironment.

## Graphical Abstract

**Graphical Abstract:**
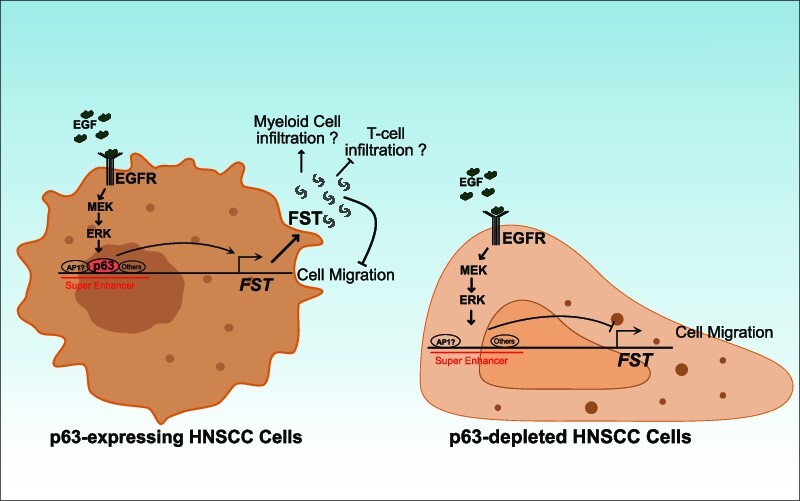

## INTRODUCTION

Cancers derived from the mucosal epithelia of the oral cavity, pharynx and larynx, collectively known as head and neck squamous cell carcinoma (HNSCC), continue to contribute significantly to global cancer statistics (1,2). Risk factors for HNSCC include alcohol and/or tobacco consumption and human papillomavirus infection. The etiological, biological and clinical heterogeneities underlying this disease poses major challenges for effective and targeted treatment. Patients afflicted with HNSCC are often not diagnosed until the disease is at an advanced stage, necessitating multimodal treatment plans, including surgery, radiotherapy and chemotherapy (3). Despite great consideration for tumor localization and disease stage, current approaches for HNSCC yield relatively poor therapy outcomes, as they overlook the underlying etiology and molecular heterogeneity of this malignancy (4). Hence, the identification of prognostic biomarkers and drivers of HNSCC is critical for early disease detection and targeted treatments.

Large-scale genomic studies have begun to reveal the molecular complexity of HNSCC, including intra- and inter-tumor heterogeneities as well as shared gene expression profiles with other cancers of epithelial origin (5–7). These studies highlight the presence of tumor suppressor inactivating mutations, activating oncogenic mutations and more recently, oncogenic fusion events (5,8,9), however it is likely that additional molecular events further contribute to oncogenic processes. Hence, continued leveraging of the genomic and genetic landscape of HNSCC might identify novel targets, and guide the development of more effective therapies. In this regard, exploration of the Transcription Factor (TF) networks as direct or indirect targets for therapeutic intervention, as well as the potential of specific TFs as biomarkers for better predicting and monitoring treatment response might be of use. One such pivotal candidate TF is tumor protein p63 (p63) which directs the squamous phenotype in HNSCC, a role that also extends to other epithelial cancers such as non-small cell lung cancer and a subset of aggressive pancreatic and bladder cancers (10–15).

As the master regulator of epithelial development, p63 is responsible for the renewal and lineage commitment of stem cells that form epithelium-rich tissues and organs (16–19). This deterministic role of p63, primarily driven by the ΔNp63 isoform, also extends to squamous carcinomas, in which the actions of p63 are amplified through oncogenic signaling pathways that control a broad range of target genes (20–23). The interaction of p63 with its cofactors and chromatin modifiers further expands its sphere of influence to practically every hallmark of cancer (24–28). Nevertheless, many aspects of p63 biology, including its full repertoire of downstream targets and their effect on HNSCC development and progression remain to be elucidated.

To uncover these targets, we performed an integrative patient-centered analysis using bioinformatics-driven data mining of the Cancer Genome Atlas (TCGA) dataset. When combined with a comprehensive RNA-seq and ChIP-seq analysis of representative HNSCC cell lines, our analysis allowed us to generate a global p63 cistrome and a disease-relevant broad oncogenic gene signature. Specifically, we leveraged p63-associated gene expression profiles of HNSCC tumors compared to normal tissue adjacent to the tumor and the super-enhancer landscape of HNSCC cell lines to identify and short list a number of known and novel oncogenes and tumor suppressors that are likely to be key mediators of p63 function. Our study highlights several genes in the EGFR and TGF-β signaling pathways as key constituents of the p63-gene signature that can stratify tumors from normal tissues in an independent cohort of HNSCC patients. We identified Follistatin (FST), a secreted glycoprotein and inhibitor of transforming growth factor TGF-β/Activin signaling pathways, as a p63 target in the epithelial component of the tumor microenvironment (TME) that might play a role in directing stemness and metastasis in HNSCC. Interestingly, our analysis also revealed a hitherto unreported role for both p63 and the epidermal growth factor receptor (EGFR) signaling pathway in driving epithelial FST expression in HNSCC. Finally, we probed bulk and single cell RNA-seq HNSCC datasets to establish the cell-type specific enrichment of *FST* expression and discovered a possible link between the p63-EGFR-FST axis and the immune component of the HNSCC TME. Taken together, our studies offer new mechanistic insights into the p63 regulatory network that is operational in HNSCC and identifies FST as a predictive biomarker and an attractive potential candidate for therapeutic targeting in HNSCC.

## MATERIALS AND METHODS

### Cell culture

SCC25 cells were grown in Dulbecco's modified eagle medium with nutrient mixture F-12 supplemented with 1% hydrocortisone. A253 cells (a gift from Jill Kramer, University at Buffalo) were grown in McCoy's 5A modified medium. CAL27 cells, were obtained from ATCC and cultured in Dulbecco's modified Eagle's medium. All growth media were supplemented with 1% penicillin/streptomycin (100 IU/ml) and 10% fetal bovine serum and replaced every 48 h. Once the cultures reached 80% confluency, cells were detached with TrypLE (Gibco Life Technologies) and passaged. All cell lines used in the study were authenticated by short tandem repeat profiling and periodically tested for mycoplasma contamination.

### Growth factor treatment and ERK inhibitor experiments

Cells were serum-starved for 24 h prior to the addition of growth factors. For initial experiments we used 50 ng each of epidermal growth factor (EGF, Cat: 236-EG), Activin A (Cat: 338-AC-010), Bone Morphogenic Protein (BMP7, Cat: 254-BP-010) and transforming growth factor beta 1 (TGFβ1, Cat: 240-B-002) (all growth factors were obtained from R&D Systems). Subsequent experiments used lower concentrations of EGF as indicated in the figure legends. Cells were incubated at 37°C for 2, 4 or 6 h with growth factors before they were processed for protein extraction. ERK1/2 inhibition was accomplished by treating cells grown in regular media with Selumetinib, ADZ6244 (Selleckchem) for 24 h. To block EGF-mediated ERK1/2 activation, cells were treated with ADZ6244 for 15–30 min prior to the addition of 10ng EGF, following which the cells were incubated for an additional 6hrs before collection and protein extraction.

### Lentiviral mediated knockdown and overexpression

Lentivirus-mediated depletion of p63 was performed using the pGIPZ system as previously described (29). pGIPZ lentiviral small hairpin RNA (shRNA#1; clone ID pV2LHS_201706; shRNA#2; clone ID pV2LHS_199988) targeting *FST* was obtained from gene modulation core facility at Roswell Park, Buffalo, NY. The lentiviral particles were packaged in HEK-293T cells by using a trans-lentiviral shRNA packaging system and harvested from the cell supernatant 48 h later. Target cell lines were transduced with the virus in the presence of 4 μg/ml Polybrene to aid transduction efficiency. Stably transduced target cells were selected using 2 μg/ml puromycin. Stable transduction was also confirmed via green fluorescent protein (GFP) expression.

A plasmid harboring the full-length human *FST* transcript variant 344, and an in-frame 3′ FLAG tag in the pcDNA3.1+/C-(K)-DYK vector was obtained from GenScript. The *FST* cDNA was excised from its parent plasmid and subcloned into the EcoRI-Xho1 site in the pLEX vector (Open Biosystems) for stable expression of FLAG-tagged FST in target cells. The *FST* transcript variant 344 encodes the 315 kDa FST isoform (FST 315) which is more relevant to our study due to its high expression in cancer cells.

### Clonogenic assay

HNSCC cells were seeded in 6-well plates at a density of 500 cells/well and maintained for 9 days at 37°C and 5% CO_2_. The cells were fixed for 2 h in an acetic acid-methanol solution (1:3 [vol/vol]), washed twice with phosphate-buffered saline (pH 7.0) and stained with 2% (wt/vol) methylene blue in 50% ethanol for 20 min. Excess stain was rinsed off with tap water, and the plates were air dried before imaging and cell counting.

### Spheroid formation and migration analysis

HNSCC cells were seeded at a density of 5000 cells/well in a 96-well ultra-low attachment plate (catalog number 7007; Corning, Corning, NY). The cells were pelleted by centrifuging the plates at 1250 rpm for 3 min and then incubated for 24–72 h (37°C and 5% CO_2_) to obtain single self-organizing spheroids in each well. The spheroids were then transferred to 35-mm culture dishes or 24-well plates where they were imaged and measured (using ImageJ) and returned to the incubator. After 96 hours, the spheroids were imaged again and the area covered by the migrated cells was determined by dividing the area covered by migrated cells by the area covered by the original spheroid.

### Invasion assay

Cell invasion assays were carried out using the Corning^®^ BioCoat™ Matrigel^®^ Invasion Chamber system (Corning), according to manufacturer protocol. Briefly, approximately 50,000 cells in serum-free media were seeded into 24-well plate transwell inserts in triplicate. The inserts were then carefully lowered into wells with media containing serum (as chemoattractant). The cells were allowed to incubate at 37°c for 24 h for A253 cells and 48 h for SCC25 cells. Following this, the unmigrated cells were removed with moist cotton swabs, while the migrated cells were fixed using 100% methanol and stained with 2% methylene blue. As an alternative approach, the fixation step was omitted and the GFP-signal from the cells was imaged using a fluorescent microscope.

### RNA isolation and sequencing

Total RNA from cells was purified using a Direct-zol RNA Mini-prep kit (Zymo Research) according to the manufacturer's instructions. The purified RNA was snap-frozen on dry ice and stored at −80°C before library preparation. cDNA libraries were prepared using the TruSeq RNA sample preparation kit (Illumina) and sequenced on an Illumina HiSeq 2500 sequencer. Quality control metrics of the raw sequencing reads were determined using FASTQC v0.4.3. Reads were mapped to the reference human genome (GRCh38/hg19 build) with HISAT v2.1.0 (30–32). Reads aligning to the reference genome were quantified with featureCounts v1.5.3 (33) to generate a matrix of counts, with columns corresponding to samples and rows containing the genes names. This was then imported into R (v 3.4) for further processing to generate normalized expression values in transcripts per million (TPM) values according to the method proposed by Wagner *et al.* (34). Differential gene expression analyses comparing control and test conditions were carried out using DESeq2 (v1.24.0) (35). Differentially expressed genes (DEGs) with a false-discovery rate value of **≤**0.1 were considered statistically significant.

### TCGA correlation analysis and derivation of gene signature

TPM-normalized values for both normal and tumor samples of the TCGA-HNSCC data set were retrieved from the Gene Expression Omnibus (GEO) website using the accession number GSE62944 (36). Pairwise correlation analysis was performed using the Hmisc package v5.0–1 in R, by comparing the expression of p63 across the normal and cancer datasets to every other gene in the matrix. A subset (*n* = 44) of the TCGA-HNSC cancer dataset for which matched normal gene expression samples were available was used for this analysis. Next, differential gene expression analysis was performed by comparing the 44 normal samples to the 502 cancer samples using DESeq2 v 1.36 (35). Significant genes were determined at an adjusted *P*-value of ≤0.1. The overlap between both analyses was determined by identifying genes that were either upregulated in cancer and showed positive correlation with p63 or were downregulated and showed negative correlation with p63 expression. The resulting dataset was then subjected to a final round of filtering to identify genes that showed corresponding upregulation or downregulation following the depletion of p63 in HNSCC cell lines. The final 430-gene p63 signature was then used as input for unsupervised hierarchical clustering and stratification of an independent large-scale HNSCC dataset generated by Huang *et al.* (37) (http://www.linkedomics.org/data_download/CPTAC-HNSCC/).

### HNSCC patient survival analysis

The R package RTCGA.clinical (v 3.5) was used to obtain the clinical data corresponding to HNSCC patients. The clinical data were merged with the expression data and ranked by decreasing expression of the genes of interest. The ‘surv_cutpoint’ function from the survminer (v0.4.9) R package (38) was used to determine the outcome-oriented optimal cut point between patients with high and low *FST* expression and to generate the survival plot.

### ChIP-seq analysis

HNSCC cells were processed for chromatin immunoprecipitation sequencing (ChIP-seq) experiments as previously described (29). Briefly, cells were grown to 80% confluency in 150-mm culture dishes and cross-linked with 1% formaldehyde for 10 min. 200–500 bp chromatin fragments were generated by subjecting the cells to four cycles of sonication. Immunoprecipitation of chromatin complexes was accomplished using the following p63 antibodies: (2 μg polyclonal antibody ΔNp63 1.1 gift from Dr Borivoj Vojtesek (39)); (1 μg polyclonal antibody H129, Santa Cruz) and (2–3 μg monoclonal antibody 4A4 (40)). Sequencing libraries were generated from DNA purified from immunoprecipitated chromatin complexes using TruPlex DNA-seq kit from Rubicon. Libraries were sequenced on an illumina HiSeq 2500 machine. The sequencing reads from all experiments were mapped to the *Homo sapiens* genome (hg19 build) using Bowtie v1.1.1 (41) with the parameter m = 1 to remove all reads mapping to multiple genomic loci. Peak calling was then performed using MACS2 v2.0.10 (42,43). Irreproducible discovery rate (IDR) analysis was used to identify consensus regions across the different p63 experiments. The resulting ChIP-seq Peaks were annotated by mapping them to the nearest gene using default settings on the Genomic Region Enrichment Annotation Tool (GREAT) v4.04 (44). Visualization of ChIP-seq peaks was done using the Integrative Genomics Viewer (Broad Institute), and bigwig files used as input were generated from Chip-seq binary alignment module (bam) files using deeptools v3.3.2 (45).

### Histone ChIP-seq clustering analysis

The fluff (46) heat map function was used to process enrichment signals from the histone ChIP-seq bam files. The signals were centered on p63 binding sites identified by IDR analysis, and clustered using *k*-means clustering. The resulting output matrix file containing the cluster assignments was then used as input for the fluff bandplot function to generate the three cluster profiles.

### Microarray analysis

The R Bioconductor package affy (v1.62.0) was used to download and normalize the microarray datasets GSE31056 (47) and GSE30784 (48) from the GEO. The normalized data were used as input to compare gene expression in normal and cancer tissues.

### Single-cell sequencing analysis

Single-cell data from Puram *et al.* (49) were downloaded from GSE103322. The data were preprocessed using the Bioconductor Seurat package (v4.1.1) (50) as previously described (51) but with the following modifications. First, the entire data set, including data from nonepithelial cells, was used for analysis. Second, variance stabilization and data normalization were performed using the sctransform function (52) in Seurat package. Dimension reduction by principal component analysis (PCA), and uniform manifold approximation and projection (UMAP) clustering were performed as part of the workflow. Determination of cluster identity was performed using Bioconductor SingleR package (53). Finally, the identity of each cluster was verified using the marker genes reported in the original study.

### Gene set enrichment analysis

Gene set enrichment analysis was performed using either Metascape (54) or the website tool from the Broad Institute (55) as indicated in the figure legends. Briefly, the DEGs were grouped into upregulated and downregulated genes and separately supplied as queries to the gene set enrichment tool. The resulting annotation was then further processed in R or Affinity Designer to promote readability.

### Protein–protein interaction network analysis

A protein–protein interaction network (Figure 3C) was generated by using the 293-target signature gene list as the input for the STRING (v11.4) webtool (available at string-db.org). The resulting network was then ported into the Cytoscape app (56,57) by using the automation pipeline plugin on the STRING website. Next, the gene expression data consisting of 293-targets were mapped onto the network to generate a color code that reflects the changes in expression, and the network was rearranged using the ‘yfiles tree layout’ module to reveal the underlying structure within the network.

### Western blot analysis

Whole-cell lysates from cells grown to 80–90% confluency was prepared in Laemmli sample buffer (Bio-Rad). The samples were heated to ∼100°C for 10 min to denature the proteins before fractionation by SDS-polyacrylamide gel electrophoresis. Proteins were then electro-transferred to Immunoblot polyvinylidene difluoride membranes (Bio-Rad), which were incubated with the following antibodies: p63 4A4, FST (sc-365003 [Santa Cruz Biotechnology] or EPR10903 [Abcam]), pAKT ser-473 (66444-I-Ig; Proteintech), pAKT Thr-308 (29163-1-AP; Proteintech), pSTAT3 (Epitomics), STAT3 (2281-1; Epitomics), AKT1 (10176-2-AP; Proteintech), pERK1/2 (sc-7883; Santa Cruz Biotechnology), ERK1/2 (sc-66192-1-Ig [Proteintech] or sc-514302 [Santa Cruz Biotechnology]), GAPDH (MAB374; EMD Millipore), pSMAD2 (GTX13364; GeneTex), SMAD2 (GTX111075; GeneTex), pSMAD1/5 (41D10; Cell Signaling), and SMAD1 (D49D7; Cell Signaling).

### Statistical analysis

Statistical analyses were performed either using R or Microsoft Excel (Microsoft Inc.). Data reported within this manuscript are presented as means ± standard deviations. All experiments were performed at least in triplicates, and statistical significance was determined using two-tailed Student's *t* tests.

## RESULTS

### p63 expression correlates with a broad epithelial and differentiation program in HNSCC

To define the transcriptional network associated with p63 in HNSCC (Figure S1A in the supplemental material), we first identified genes that significantly correlate with p63 expression by using the TPM-normalized TCGA HNSCC data set from GSE62944 (36). As a first step, we generated pairwise correlation scores for the expression of each gene in relation to p63 expression in cancer and normal tissues using data from the 44 patients with matched normal samples. The inclusion of gene expression values from the normal adjacent tissues, enabled us to set a normal baseline for p63 expression in estimating correlation scores for all other genes. Of the 22565 genes profiled, a large percentage (73%) correlated positively with p63 (Supplementary Table S1), suggesting that p63 acts primarily as a transcriptional activator.

We next compared the transcriptomes of the 44 normal adjacent tissue samples to those of the entire set of 504 HNSCC samples to identify the p63-correlated genes that are differentially expressed between normal and cancer tissues. This analysis identified 14912 differentially expressed genes (DEGs) at a false discovery rate of ≤10% (adjusted *P* value ≤ 0.1). A comparison of the log2 fold change values of the DEGs with their correlation scores identified 11970 genes that changed in the same direction as that predicted by the p63 correlation scores, i.e. the genes were either upregulated and had positive correlation scores or downregulated and had negative correlation scores (Supplementary Table S1).

We reasoned that these correlated DEGs are likely to be involved in p63-regulated tumorigenic processes in HNSCC. Accordingly, gene ontology (GO) analysis of the top 1500 positively correlated DEGs showed an enrichment in biological processes such as proliferation, cell division and DNA replication (Supplementary Figure S1B). Conversely, the genes that were downregulated and negatively correlated with p63 were enriched in differentiation processes such as keratinization, osteogenesis, myogenesis and organelle organization (Supplementary Figure S1C). These results are consistent with the established role of p63 in the maintenance of stem cells and suppression of differentiation in HNSCC (24,28). To further explore the biological relevance of these genes, we performed pathway analysis which revealed that the positively correlated DEGs were associated with mRNA processing, DNA damage response and TGF-β pathways (Supplementary Figure S1D), whereas the negatively correlated DEGs were associated with various metabolic and functional pathways, such as lipid and fatty acid metabolism and muscle contraction pathways (Supplementary Figure S1E).

Given the large number of DEGS identified in both screens, we suspected that our list may represent both bona-fide targets of p63, as well as genes that are simply a reflection of the profound variation inherent in the tumor samples.

### Integrative analysis of HNSCC patient and cell line datasets defines a p63 signature

**Figure 1.**
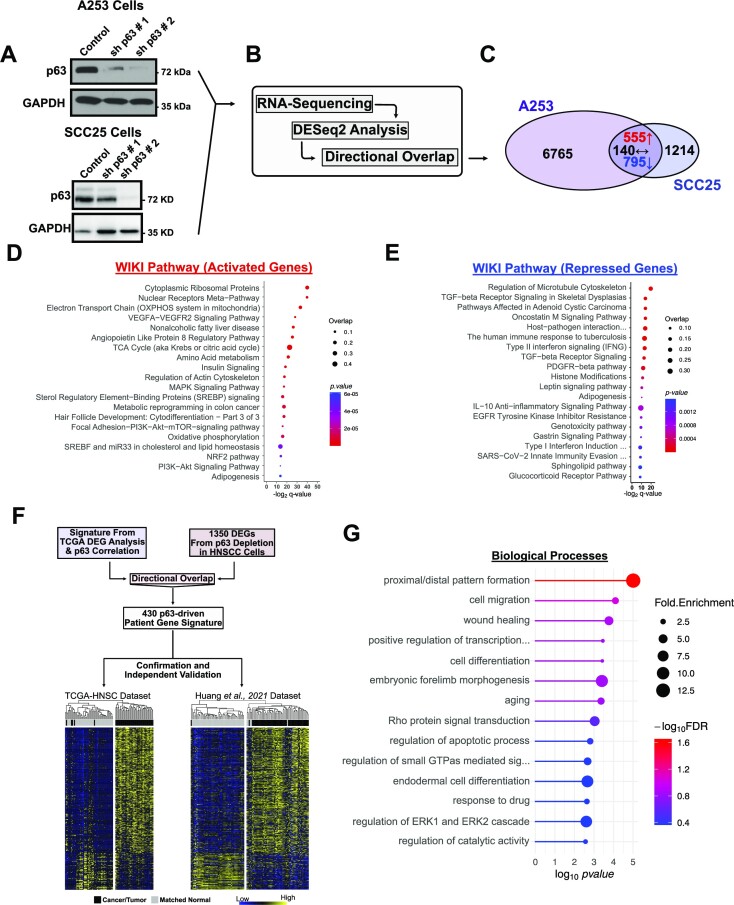
p63-driven gene signature. (**A**) Western blot showing robust depletion of p63 in A253 and SCC25 cells treated with p63-targeting shRNA constructs. (**B**) Schematic showing the analysis of the RNA-seq data generated from the p63 knockdown experiments. (**C**) Venn diagram showing the numbers of p63-regulated DEGs in both A253 and SCC25 cells. 555 genes (red) were upregulated, and 795 genes (blue) were downregulated in both cells. 140 genes (black) were not consistently upregulated or downregulated in both cells. (**D**) WikiPathway analyses of the genes that were upregulated following p63 (**E**) WikiPathway analyses of the genes that were downregulated after p63 depletion. (**F**) (Top) Schematic showing how both TCGA-HNSCC patient data and *in vitro* knockdown data were combined to generate the consensus signature. (Bottom left) Heat map showing the expression profile in the 430-gene consensus signature in the TCGA data set. (Bottom right) The pattern of expression of this signature is conserved in an independent preclinical HNSCC data set reported by Huang *et al.* (37). (**G**) Biological processes enriched in the 430-gene consensus signature, including Rho and ERK1/2 signaling cascades, cell migration and cellular response to drugs.

To evaluate the transcriptomic events specifically regulated by p63, we used lentiviral-based delivery of shRNA to deplete p63 expression in two well-established HNSCC cell lines, A253 and SCC25 (58–61). We confirmed diminished p63 expression by two independent shRNAs via western blot analysis in both cell lines (Figure 1A), and performed RNA-sequencing to determine the global changes in gene expression. We identified ∼8200 differentially expressed genes (DEGs) in A253 cells and 2700 DEGs in SCC25 at a false discovery rate (FDR) of 10% (Supplementary Figure S2A, B). A GO analysis of these DEGs indicated that p63 may promote stemness in cells by repressing genes involved in cellular differentiation (Supplementary Figure S2C, D) while favoring proliferative processes such as transcription, growth factor signaling and energy production (Supplementary Figure S2E, F). This result is well in agreement with the known p63 oncogenic program in SCC cells (24,28).

To identify a consensus p63-mediated transcriptome, we determined the DEGs common to both A253 and SCC25 cells (Figure 1B). This analysis identified 1350 genes (Figure 1C), which were then used to perform a pathway analysis. The upregulated genes were enriched in hair follicle development, insulin signaling and lipid and cholesterol metabolism (Figure 1D), whereas the downregulated genes were mostly involved in TGF-β, growth factor and interferon signaling (Figure 1E), similar to the results from the analysis of patient datasets.

Given the concordance of the p63 regulated processes generated from our *in-vitro* cell line models, we proceeded to integrate the results from the cell-line based analysis (1350 genes) with the results from the HNSCC patient analysis (11 790 genes) to identify a common p63 gene signature. We found that 430 genes that correlated with p63 expression across normal and cancer tissues, were also dysregulated following the loss of p63 expression in both A253 and SCC25 cells (Figure 1F & Supplementary Table S1). To determine whether this p63 consensus gene signature can be used to discriminate between normal adjacent tissues and tumors, we performed unsupervised hierarchical clustering on a combined matrix of the data from 44 HNSCC and normal adjacent tissues as well as an independent human papillomavirus HPV-negative HNSCC data set generated by Huang *et al.* (37) from 53 normal adjacent tissues and 109 tumor samples. Our p63 consensus gene signature segregated most of the cancer and normal adjacent tissue samples in both datasets (Figure 1F), suggesting that these genes are critical drivers of the oncogenic processes in HNSCC. Notably, a GO analysis showed that the p63-driven consensus gene signature is enriched for biological processes such as migration, differentiation and signal transduction processes (Figure 1G).

### Global genomic occupancy by p63 in SCC cells identifies direct transcriptional targets

To establish a mechanistic basis for the observed changes in gene expression driven by p63, we next performed ChIP-seq experiments in both A253 and SCC25 cells using p63-specific antibodies. Pairwise correlation analyses on the replicates for each cell line revealed good agreement in both the number and quality of p63 binding sites identified by two p63 antibodies (Supplementary Figure S3A). We also used the irreproducible discovery rate to measure the consistency between ChIP-seq replicates and identified 13800 and 37516 sites in A253 and SCC25 cells, respectively (Supplementary Table S2).

A *de novo* motif analysis using the MEME-ChIP suite (62,63) confirmed the core p63 motif was the most enriched in both cell lines (Supplementary Figure S3B). We used the TOMTOM program (64) to compare the *de novo* analysis motif against the human transcription factor database HOCOMOCO (65), which confirmed that our enriched core motif is identical to the p63 motif (*P =* 4.5e-360). In addition to the core motif, this analysis also identified several other enriched TF motifs, including those for the AP-1, KLF and FOX family (Supplementary Figure S3B), suggesting that these TFs may act as transcriptional cofactors of p63. We also used the ChIPseeker program (66) to evaluate the genomic distribution of p63 peaks relative to transcriptional start sites; p63 binding sites were enriched at distal and intragenic regions (Supplementary Figure S3C), which is consistent with the known predilection of p63 to target enhancers (67).

To better understand the p63 regulatory landscape, we performed follow-up ChIP-sequencing analysis with both cell lines for three epigenomic marks: acetylation of lysine 27 on histone 3 (H3K27ac), which marks active regulatory regions, and mono-methylation and trimethylation of lysine 4 on histone 3 (H3K4me1 and H3K4me3, respectively), which mark enhancer and promoter targets, respectively (68). K-means clustering of normalized levels of histone modifications at each p63-bound region indicated that approximately 7.9% and 5.6% of p63 binding sites were associated with the promoter H3K4me3 mark in A253 and SCC25 cells, respectively (Supplementary Figure S3D). Surprisingly, less than 23% of the enhancer-associated p63 binding sites were marked by robust H3K27ac. By contrast, the largest proportion of p63 binding was associated with poised enhancers, with strong H3K4me1 marking but weak H3K27ac marking (Supplementary Figure S3D).

GO annotation of the genes associated with the regulatory regions, as determined via GREAT (44), revealed that the different clusters are associated with genes involved in distinct biological processes (Supplementary Figure S3E). The cluster representing the poised enhancers was associated with genes involved in various differentiation processes such as branching morphogenesis, keratinocyte differentiation and cornification. The cluster representing active promoters (strong marking for H3K27ac and H3K4me3) was associated with genes involved in developmental processes, such as epidermis development, extracellular matrix organization, wound healing and apoptosis. The cluster representing active enhancers (strong marking for H3K27ac and H3K4me1) was associated with genes involved in the cell cycle and cell–cell/matrix adhesion processes. Together, these results point to an oncogenic role for p63 in which it drives the cellular proliferation program at the expense of differentiation.

### P63 occupies super enhancers more frequently than regular enhancers in HNSCC

**Figure 2.**
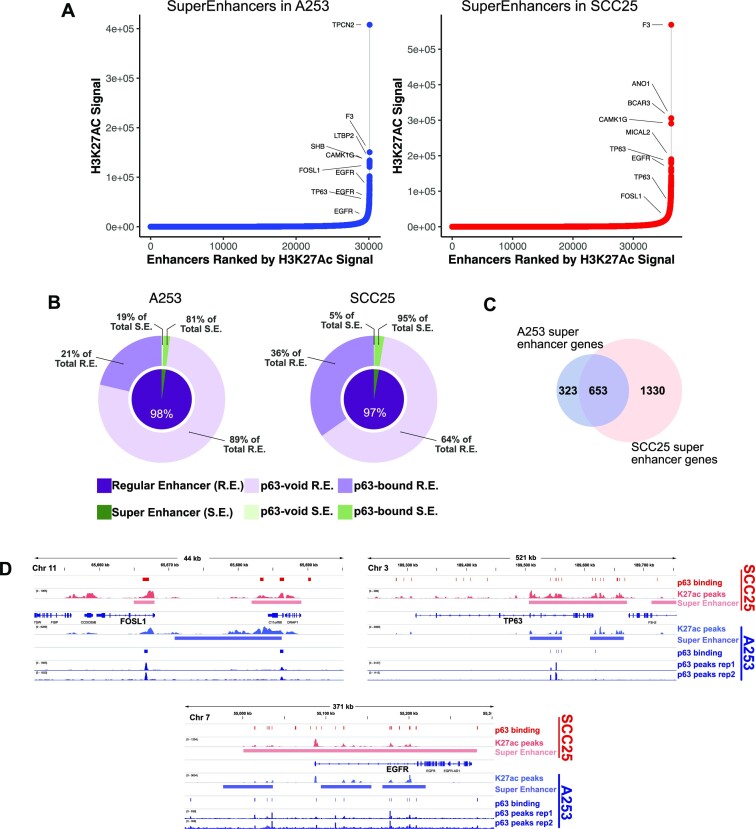
Overview of the super enhancer landscape in A253 and SCC25 cells. (**A**) Super enhancer profiles of A253 and SCC25 cells according to H3K27Ac with gene assignment. (**B**) Pie charts detailing the distribution of p63 binding in regular enhancer versus super enhancer regions. (**C**) Common super enhancer-associated genes in A253 and SCC25 cells. (**D**) Genomic maps showing the super enhancers associated with TP63, FOSL1 and EGFR in both A253 and SCC25 cells.

The presence of an active enhancer cluster prompted us to evaluate the possibility that p63 binds super enhancers, which are large clusters of enhancers that control the expression of cell identity genes and are enriched for lineage-specifying and oncogenic transcription factors, such as p63 (69,70). We mapped the super enhancer landscape by implementing the ROSE algorithm (70,71) on H3K27ac ChIP-seq data from both HNSCC cell lines and identified 642 super enhancers in A253 cells, and 963 super enhancers in SCC25 cells which is in agreement with our prior results (60) (Figure 2A). We identified the associated genes by mapping each super enhancer to their nearest genes using GREAT.

Interestingly, most of the super enhancers were bound by p63 in both cells, while only small proportion of the regular enhancer regions were associated with p63 binding (Figure 2B). There were 653 genes associated with super enhancers that were common to both cancer cell lines (Figure 2C), including oncogenes known to regulate aspects of HNSCC biology. For example, *TP63*, *EGFR* and *FOSL1*, known regulators of stemness and malignancy (72–74), were associated with super enhancers encompassing multiple p63 binding sites in HNSCC (Figure 2D). These results provide further evidence for a role for p63 in driving an oncogenic gene expression program in HNSCC.

### P63 directs a highly interconnected EGFR–TGF-β1 network in HNSCC

**Figure 3.**
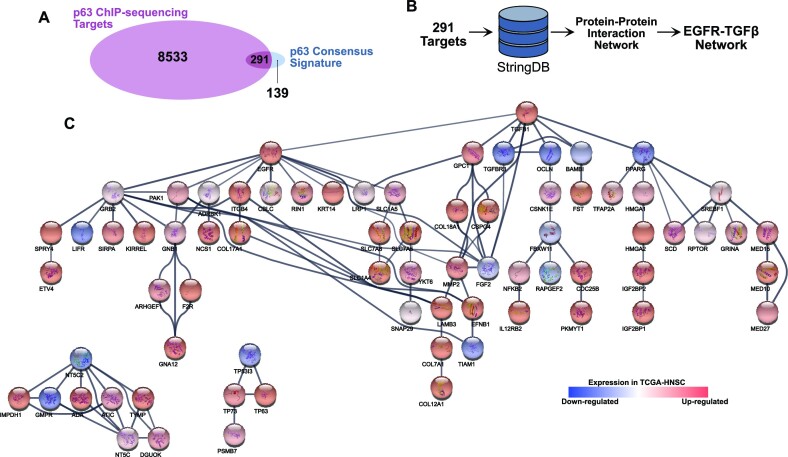
Protein interaction network involving EGFR and TGF-β signaling pathways embedded in the p63 signature network. (**A**) Venn diagram showing the 291 genes that overlap between the p63 targets and p63 consensus signature. (**B**) The 291 genes were used as input to perform an interaction network analysis on the STRING database (75–77). (**C**) Approximately 21% (61) of the 291 targets used as input in the STRING database were part of an interconnected network of protein–protein interactions that link the TGF-β1 signaling and EGFR signaling. The bubbles are color coded to reflect the corresponding log_2_ fold change values.

We next sought to determine if the super enhancer-associated genes were part of the p63 consensus signature that was identified. We mapped the p63 binding sites from both A253 and SCC25 cells to their putative targets using the default (basal plus extension) setting for the GREAT and identified 8824 targets common to both cell lines. A large proportion (68% [291/430]) of the genes in the p63 consensus signature were associated with a p63 binding site (Figure 3A). We therefore posited that there may be functional interactions among the genes in this target signature. To test this, we queried for a protein–protein interaction network and performed functional enrichment analysis using the STRING database (75–77) (Figure 3B). This revealed an extensive interconnected network of predicted interactions involving 61 of the 291 targets genes in the p63 consensus signature; the network included key molecules in the epidermal growth factor receptor (EGFR) and TGF-β1 signaling pathways (Figure 3C).

**Figure 4.**
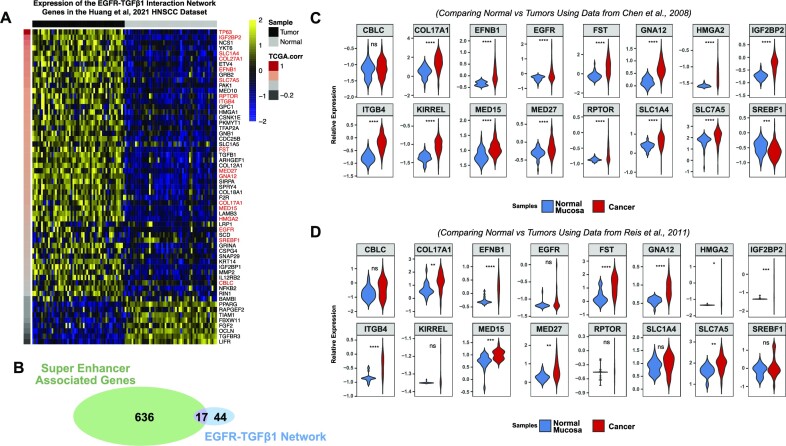
EGFR–TGF-β1 network signature is conserved in multiple HNSCC datasets. (**A**) Heat map showing the expression of the 61-gene EGFR–TGF-β1 signature in the HNSCC data generated by Huang *et al.* (37). (**B**) Venn diagram showing overlap of 17 genes between the super enhancer-associated and EGFR–TGF-β1 signatures Expression of the 17 common genes in (**C**) the oral carcinoma microarray data set reported by Chen *et al.* (48), and in (**D**) the HNSCC microarray data set reported by Reis *et al.* (47). Statistical significance was determined using unpaired Student's *t* tests: *P < 0.05, **P < 0.01, ***P < 0.001, ****P < 0.0001.

The expression pattern of genes in the network was consistent with that in the independent data set generated by Huang *et al.* (37) (Figure 4A), suggesting that these genes may be critical for HNSCC biology. In a parallel analysis, we overlaid the 653 super enhancer associated genes with the genes present in our newly identified EGFR–TGF-β1 network signature and identified a subset of 17 genes that are common to both lists (Figure 4B and highlighted in red in Figure 4A). Among the genes in this subset were several stem cell markers, such as *ITGB4*, *HMGA2*, *IGF2BP2*, *COL17A1* and *FST* (78–81). To further validate the functional importance of these genes, we examined their expression in two separate HNSCC microarray datasets (47,48), which revealed *ITGB4*, *FST*, *GNAI12* and *EFNB1* to be among the genes showing significant upregulation in both datasets examined (Figure 4C & D).

### Follistatin is a novel target of p63 in HNSCC

Given the consistent overexpression of *FST* across multiple HNSCC datasets, and the enrichment of the TGF-β network in our analysis, we reasoned that FST may play an important role in the oncogenesis of HNSCC. FST is a secreted glycoprotein that binds and neutralizes the ligands of the TGF-β superfamily, and has been shown to play a role in regulating the development, progression and metastatic dissemination of epithelial cancers (82–84). Although a role for the p63-FST axis in the regulation of Activin signaling has been shown in mouse salivary glands (81), a relationship between p63 and FST has not been established in HNSCC.

To confirm whether the p63-driven regulation of FST is conserved in HNSCC, we evaluated FST expression in control and p63-depleted A253 and SCC25 cells. The results showed the loss of FST expression following p63 depletion in both cell lines (Supplementary Figure S4A), in agreement with the RNA-seq results that revealed FST to be a likely transcriptional target of p63 (Figure 3C). To probe this further, we examined the *FST* genomic locus using our ChIP-seq data and identified several p63 binding sites within a super enhancer cluster located ∼300 kb from the *FST* gene (Supplementary Figure S4B). Taken together, these results identify p63 as an upstream oncogenic driver of *FST* expression in HNSCC.

### P63 mediates EGFR signaling to drive FST expression in HNSCC

**Figure 5.**
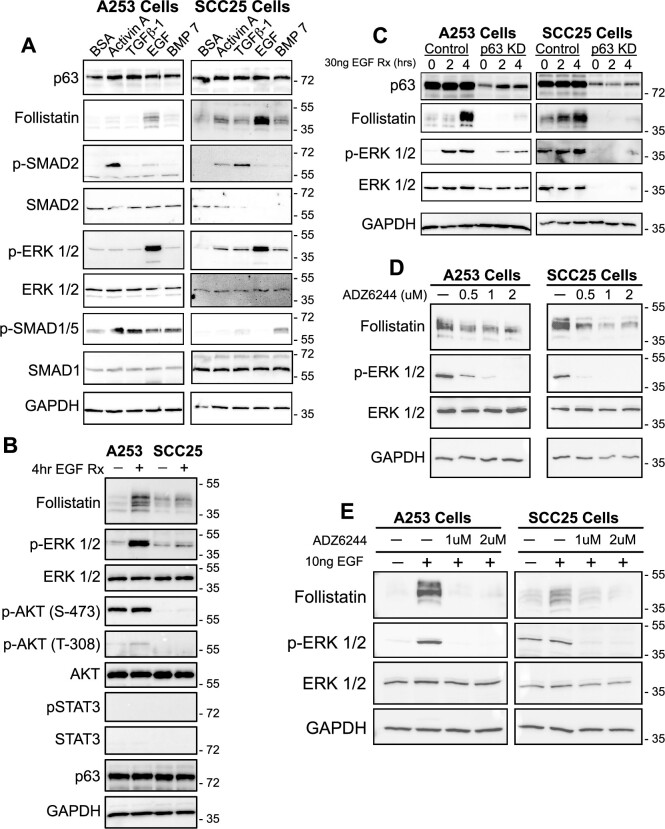
EGF signaling drives FST expression via ERK signaling in HNSCC. (**A**) Western blots showing the effects of 4-h stimulation of SCC25 (left) and A253 (right) cells with 50ng of indicated growth factors. Although the downstream signaling cascades of these growth factors were activated in both cell lines, only EGF treatment upregulated FST expression. (**B**) Treatment of A253 and SCC25 cells with 30 ng EGF for 4 h resulted in activation of ERK1/2 but not AKT or STAT3. (**C**) Depletion of p63 prevented EGF-mediated FST expression in A253 and SCC25 cells. (**D**) Inhibition of ERK phosphorylation via Selumetinib (ADZ6244) reduced basal levels of FST expression. (**E**) Inhibition of ERK activation by ADZ6244 blocks the EGF-mediated increase in FST expression in both A253 and SCC25 cells.

Previous studies have reported that ovarian cancer cells increase FST production in response to TGF-β stimulation (84), and FST expression has been shown to coincide with the activation of the signaling cascades downstream of EGF signaling in leukemia cells (85), however, signaling events driving FST in HNSCC cells remain unknown. Given the enrichment of the EGFR-TGF-β network in our dataset, we hypothesized that HNSCC cells might regulate FST expression in response to both signaling pathways. To test this hypothesis, we first determined the specific TGF-β superfamily ligands that are expressed in HNSCC tissues using the TCGA dataset. Our analysis revealed *TGF-β1*, activin A (*INHBA*) and *BMP7* to be the top three highly expressed ligands in HNSCC (Supplementary Figure S5). We thus treated A253 and SCC25 cells with TGF-β1, activin, BMP7 or EGF for 4 h and evaluated the expression of FST. Our Western blot results showed that of all the growth factors tested, only EGF treatment led to a consistent increase in FST protein levels after four hours (Figure 5A). While these results do not rule out the contribution of other cytokines to the regulation of FST, they suggest that the EGF signaling dominates the early phase of FST regulation.

EGF signaling can be mediated by multiple downstream signaling cascades, including the extracellular signaling regulated kinases (ERK1/2), phosphatidyl-inositol 3 kinase (PI3K-AKT) or signal transducers and activators of signaling (STAT). Activation of any of these downstream molecules via phosphorylation could potentially drive the expression of FST. Therefore, to determine which of these downstream molecules drive FST expression, we examined their phosphorylation status following treatment with EGF by western blotting. As shown in Figure 5B, treatment of both A253 and SCC25 cells with EGF led to increased phosphorylation of ERK1/2, while AKT and STAT pathways did not show significant activation. Next, we evaluated the contribution of p63 to the EGF-mediated FST expression by treating both control and p63-depleted cells with EGF. Compared to control cells, p63-depleted cells showed no FST expression following EGF treatment, confirming a central role for p63 as a downstream effector of the EGF-mediated FST regulation (Figure 5C). These findings were verified in a third HNSCC cell line: CAL27 cells, where EGF treatment resulted in the phosphorylation of ERK1/2 as well as AKT and STAT3 (Supplementary Figure S7A). Importantly, p63 depletion also blocked the EGF-mediated induction of FST in CAL27 (Supplementary Figure S7B). To independently confirm a role for ERK activation in mediating FST expression, we treated A253 and SCC25 cells with Selumetinib (ADZ6244), a selective inhibitor of ERK phosphorylation. Western blot analysis revealed a dose dependent decrease of FST expression following ERK inhibition (Figure 5D). Furthermore, ADZ6244 blocked the EGF mediated FST upregulation in both cells (Figure 5E). Altogether, these results indicate a central role for the p63-EGF-ERK1/2 signaling axis in driving FST expression in HNSCC.

### FST regulates proliferation and colony formation in HNSCC cells

**Figure 6.**
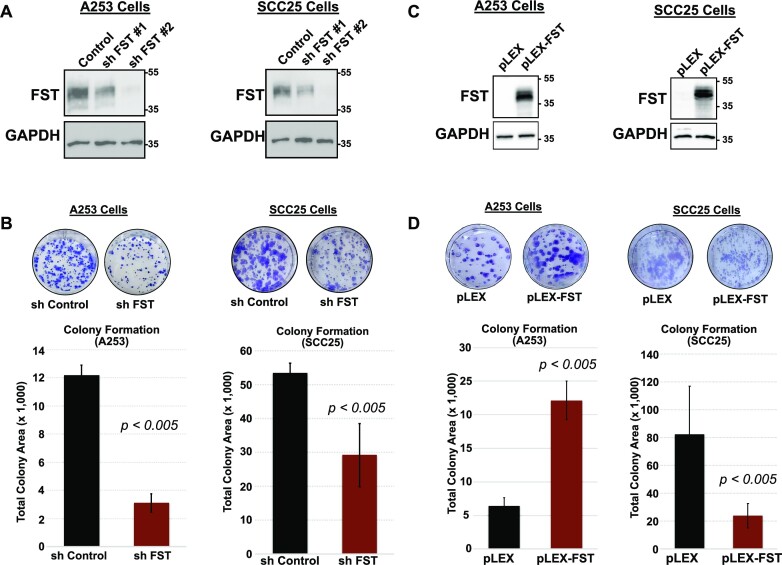
Effects of FST on HNSCC clonogenicity. (**A**) Western blot showing the depletion of FST in A253 and SCC25 cells by shRNA. (**B**) Colony formation by A253 cells and SCC25 cells after shRNA-mediated knockdown of FST. (**C**) Western blot showing lentiviral-mediated expression of FST in A253 and SCC25 cells. (**D**) Colony formation by A253 cells and SCC25 cells following overexpression of FST. Data presented as mean ± standard deviation (SD) (*n* ≥ 3). Statistical significance was determined using Student's *t* tests for paired samples.

FST has been associated with stem and progenitor cell function in tissue development (81,86,87), prompting us to consider a similar role in the oncogenic context. To investigate this, we tested two FST targeting shRNA lentiviral constructs, on both cells to examine their efficacy in depleting FST expression. Western blot analysis confirmed both shRNA constructs to be effective, with shFST #2 showing more robust depletion of FST (Figure 6A). Following the confirmation of FST depletion, we evaluated the effect of FST depletion on cell proliferation, by performing a cell proliferation assay, which showed that the loss of FST resulted in reduced proliferation in both A253 and SCC25 cells (Supplementary Figure S6A, B). Next, to determine if the loss of cell proliferation resulted in the loss of stemness, we investigated the ability of these cells to form colonies. Compared to control cells, FST depleted cells were deficient in their ability to form colonies (Figure 6B). In contrast, vector-mediated overexpression of FST (Figure 6C) enhanced colony formation by A253 cells but suppressed the same in SCC25 cells (Figure 6D). Together, these analyses suggest that FST regulates proliferation and colony formation in HNSCC.

### Effect of FST on invasion and migration of cancer cells

**Figure 7.**
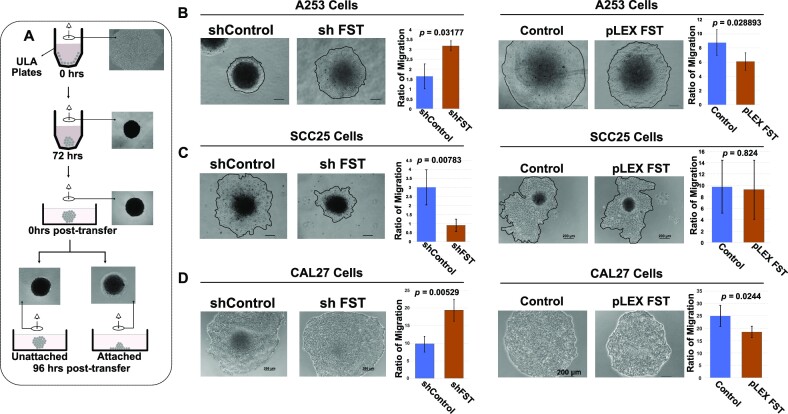
Effect of FST expression on the migration of SCC cells. (**A**) Cartoon showing the experimental setup for assessing cell migration. (**B**) (Left) Loss of FST expression enhanced the migration of A253 cells. (Right) FST overexpression suppressed the migration of A253 cells. (**C**) (Left) Loss of FST expression suppressed the migration of SCC25 cells. (Right) FST overexpression had no significant effect on the migration of SCC25 cells. (**D**) (Left) Loss of FST expression enhanced the migration of CAL27 cells. (Right) FST overexpression suppressed the migration of CAL27 cells. Data presented as mean ± standard deviation (SD) (*n* ≥ 5). Statistical significance was determined using Student's *t* tests for paired samples.

Given the role of FST as an inhibitor of TGF-β signaling, it is possible that it may influence the invasion and migration of HNSCC cells. To investigate the effect of the loss of FST on cell invasion, we subjected both A253 and SCC25 cells to matrix invasion assays using the transwell system, which revealed that the effect of the loss of FST is consistent with promoting invasion in cancer cells (Supplementary Figure S6C, D), although the results were not statistically significant in SCC25 cells. We reasoned that the effect of FST may be more pronounced in a system that closely mimics the 3D architecture of cancer cells in-vivo. To investigate this, we first grew HNSCC cells for 72 h on ultra-low attachment plates which allow them to self-organize into spheroids. The spheroids were then transferred into individual wells of a 24-well plate, to facilitate attachment and migration to form monolayers (Figure 7A). This system enabled us to assess migration by calculating the distance covered by the migrating cells, relative to the initial area covered by the spheroids (88,89). FST-depleted A253 cells showed greater migration than controls, whereas FST-overexpressing A253 cells showed reduced migration (Figure 7B). However, FST depletion reduced the migration of SCC25 cells, while FST overexpression had no significant effect on the migration of SCC25 cells (Figure 7C). Parallel studies were performed in a third HNSCC cell line, CAL27 cells, in which FST knockdown and overexpression (Figure S8A, B) produced results similar to those obtained with A253 cells (Figure 7D). Taken together, our analysis reveals that FST has a cell-intrinsic role in regulating the migration of SCC cells.

### FST expression in the tumor microenvironment is primarily restricted to epithelial cells

**Figure 8.**
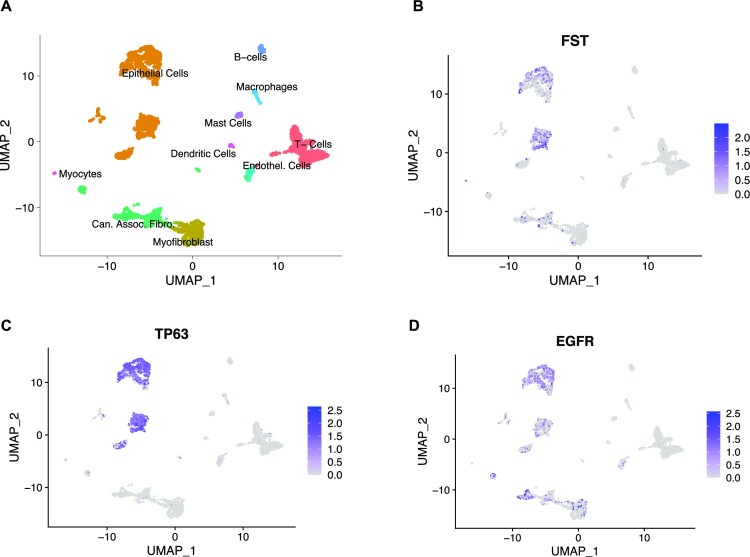
Epithelial cells are the primary producers of FST in the tumor microenvironment. (**A**) UMAP plot showing the identities of the various cellular clusters identified in the HNSCC single-cell RNA-seq data reported by Puram *et al.* (49). (**B**) Expression of *FST* by cells in the different clusters showing predominantly epithelial cell-specific expression. (**C**) UMAP plot showing that expression of *TP63* is predominantly in epithelial cells. (**D**) *EGFR* expression in the different cellular clusters.

Our analysis thus far has focused on the expression of FST by epithelial cells; however, FST could also be expressed by the myriad cell types that make up the tumor microenvironment. We reanalyzed the single-cell RNA-seq data set generated from primary HNSCC tissues by Puram *et al.* (49) to annotate the identities of the various cells within the tumor microenvironment according to markers reported for the original manuscript. We identified T cells (*CD2* and *CD3D*), fibroblasts (*MMP2* and *ACTA2*), macrophages (*CD14* and *FCGR2A*), dendritic cells (*CD83* and *CD80*), mast cells (*CMA1* and *MS4A2*), endothelial cells (*PECAM1* and *VWF*), B/plasma cells (*BLNK* and *SLAMF7*), myocytes (*ACTA1* and *MYL2*) and epithelial/cancer cells (*EPCAM* and *KRT14*) (Figures 8A and S9). We then evaluated the expression profile of *FST* across the different cellular clusters and found that *FST* transcripts were predominantly localized to the epithelial subclusters, with sparse expression in cancer-associated fibroblasts (Figure 8B). As expected, *TP63* and *EGFR* expression were also prominently enriched in the epithelial clusters (Figure 8C, D). Taken together, we conclude that epithelial cells express both p63 and EGFR and are the major source of FST within the HNSCC microenvironment.

### FST expression correlates with the immune microenvironment in HNSCC

**Figure 9.**
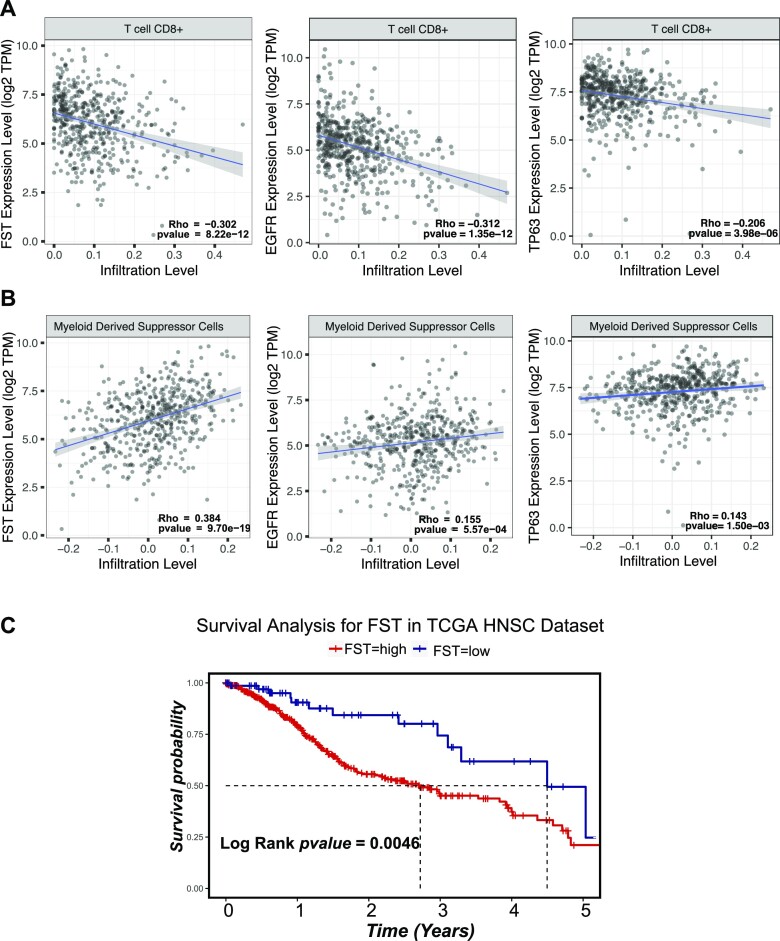
*FST*, *EGFR* and *TP63* expression correlates with immune cell infiltration. *FST*, *EGFR* and *TP63* expression correlated negatively with tumor suppressive T-lymphocyte infiltration (**A**) and positively with tumor-promoting myeloid suppressor cells (**B**) in HNSCC tissues. (**C**) Kaplan–Meier plot showing overall survival of cohorts of patients with high and low FST expression levels.

The presence of intra-tumoral immune cell infiltrates, particularly tumor-suppressive T lymphocytes, is associated with a better prognosis in HNSCC (90,91). Furthermore, EGF signaling can block the release of immunomodulatory cytokines that promote T cell infiltration in HNSCC (92), whereas pharmacological inhibition of EGFR promotes the infiltration of cytotoxic (CD8^+^) T cells in a murine model of HNSCC (93). Thus, EGF signaling may impede T-cell infiltration in HNSCC tumors and lead to poor patient outcomes. Given our findings that FST is downstream of EGF signaling, we reasoned that there may also be a correlation between FST expression and T-cell infiltration. To investigate this, we generated gene–immune cell association plots using TIMER 2.0 (94), which revealed that the expression of *FST*, *TP63* and *EGFR* correlated negatively with the infiltration of tumor-suppressive CD8^+^ T lymphocytes (Figure 9A). Conversely, we found that all three genes correlated positively with the infiltration of tumor-promoting myeloid-derived suppressor cells (Figure 9B).

On the basis of the above-described correlations, we also reasoned that FST-enriched epithelial cancer cells may influence the immune tumor microenvironment and thus affect the outcomes of patients with HNSCC. To investigate this, we stratified the TCGA-HNSCC patients into two cohorts, consisting of 398 FST-high patients, and 74 FST- low patients and investigated the prognostic value of FST by Kaplan–Meier analysis. As shown in Figure 9C, high FST expression correlated negatively with patient survival, indicating a pro-tumorigenic role of FST. Collectively these data lend credence to the idea that EGFR-p63-FST signaling in the complex tumor microenvironment of HNSCC modulates the immune response.

## DISCUSSION

Our limited understanding of the molecular etiology and complexity of HNSCC remains a barrier to effective treatment. In particular, there is a need to identify molecular biomarkers and crucial drivers of HNSCC, which can be exploited to predict disease progression, response to treatment and importantly, reveal new therapeutic targets. Given the pivotal role of p63 in the development, progression, and metastatic dissemination of various SCCs, including HNSCC, the identification and characterization of the critical downstream network of targets that mediate the oncogenic function of p63 remain a priority that is likely to yield valuable insights. We integrated transcriptomic data from TCGA-HNSCC tumors and HNSCC cell lines to define a conserved and functionally relevant p63 target gene signature. Our analyses revealed FST as a direct target of p63 and a novel marker of HNSCC oncogenesis.

Large-scale transcriptomic datasets from HNSCC patients can be leveraged to better prioritize actionable p63 target genes in HNSCC. We evaluated p63 levels and associated gene expression patterns between the tumor and adjacent normal tissues, which revealed cancer-related genes whose expression correlated with that of p63 and thus suggested a possible functional link. The use of HNSCC cell lines derived from patients enabled us to uncover the epigenetic landscape of p63-bound regulatory elements, particularly those that represent active enhancers. We posit that the H3K27Ac-enriched super enhancers we identified are associated with genes that are likely to occupy higher positions in the p63 oncogenic network. Notably, this list of super enhancer-associated genes consists of several known and emerging oncogenic factors such as *SREBF1*, which encodes a transcription factor that regulates tumor metabolism and drug resistance (95,96), and *FSCN1* and *HAS3*, two oncogenic factors that promote the growth and metastasis of tumor cells (58,97). We also identified genes that are anti-correlated to p63 expression, including putative tumor suppressors such as *EHF*, which we and others have shown to be an important regulator of tumor redox homeostasis and the immune environment (51,98), and *CXXC5*, an unmethylated CpG binder that likely acts as a nucleation factor for transcription factors, coregulatory proteins and DNA modifiers (99).

An interesting subset of the 61 genes within the p63 signature we identified was associated with an integrated EGFR–TGF-β network. Moreover, these p63-target genes showed a distinct cancer-enriched expression pattern compared to that of normal tissue in an independent primary HNSCC data set, suggesting they represent a core oncogenic signature of HNSCC biology (37). The functional link between p63 and TGF-β and other signaling pathways is exemplified by studies showing that the downregulation of p63 is required for TGF-β-induced metastasis in SCC (100), and that the loss of p63 during HNSCC development drives tumor metastasis via mitogen-activated protein kinase signaling (101). The cooperation between EGFR and TGF-β1 signaling is also an important driver of metastasis, as shown in breast cancer cells (102,103). We posit that this cooperation is similarly involved in the recently described EGF-mediated epithelial-to-mesenchymal transition in HNSCC (89). Our findings suggest that an EGFR–TGF-β signaling axis involving p63 drives HNSCC oncogenesis and that this function could partly be mediated by FST.

We uncover several interesting aspects of FST biology and function in HNSCC: first, we identified *FST* as super enhancer-associated gene in HNSCC whose expression is consistently downregulated in the absence of p63; second, we demonstrated that FST intrinsically mediates HNSCC cell clonogenicity and migration, presumably by neutralizing TGF-β/activin signaling. These observations agree with previous studies showing that depletion of activin A stops the migration of oral cancer cells (104). We postulate that the loss of p63 relieves the inhibition of TGF-β/activin signaling via FST thereby promoting tumor epithelial cell migration and metastasis, and these processes are intertwined with cross-talk between cancer cells and other key components in the TME such as fibroblasts, as in the case of prostate cancer (105). Taken together, these observations and findings offer a hitherto unappreciated pathway in which EGF-driven regulation of FST expression is mediated by p63 in HNSCC.

We found that the expression of *EGFR*, *TP63* and *FST* correlates with immune infiltration in the tumor microenvironment, such that their expression is linked not only with the absence of tumor-eliminating T lymphocytes but also with the presence of tumor-promoting myeloid-derived immune-suppressor cells. These results agree with published reports implicating tumoral EGF signaling as a major suppressor of T cell infiltration (92). Interestingly, FST drives resistance to immune checkpoint inhibitors in ovarian cancer cells (84), but future research is needed to determine whether similar mechanisms are at play in HNSCC. Nevertheless, FST represents a novel modulator of the complex EGFR–TGF-β–p63 axis and the tumor microenvironment in HNSCC. Our findings also highlight FST as a potential prognostic factor in HNSCC and that its negative effect on disease progression may result from a possible metastatic suppressor function as shown in breast cancer (82).

One limitation of our study is that we did not consider the differences in the mutational landscape or inherent molecular subtypes of the representative HNSCC cells. For example, A253 and CAL27 cells harbor mutations in the *TGFBR2* and *TGFBR1* genes respectively, whereas SCC25 cells express wildtype receptors – such differences may explain some of the discrepant functional outcomes that we observed in our studies (106,107). Future research efforts will involve a systematic and tumor subtype-specific approach to evaluating the role of FST in HNSCC. Such studies will reveal specific vulnerabilities of tumors that may lead to the development and adoption of FST blocking antibodies in the routine clinical care of HNSCC patients.

## Supplementary Material

zcad038_Supplemental_Files

## Data Availability

All sequencing data generated in this study have been deposited in the Gene Expression Omnibus (GEO) under accession number GSE212752.
