# Supplementary material for: An integrated genomic approach identifies follistatin as a target of the p63-epidermal growth factor receptor oncogenic network in head and neck squamous cell carcinoma
Source: NAR Cancer. 2023 Jul 24;5(3):zcad038. doi: 10.1093/narcan/zcad038 (PMC10365026; doi:10.1093/narcan/zcad038)
Supplement: zcad038_Supplemental_Files [file zcad038_supplemental_files.zip › Supplemental Figure Legends Revision April 2023.docx]

**SUPPLEMENTAL FIGURE LEGENDS**

**Figure S1.** p63 correlates positively with cell cycling processes in HNSCC. (A) Schematic showing the analysis for the derivation of the p63-correlated genes in the TCGA-HNSCC dataset. Biological processes of the top 1,500 positively (B) and negatively (C) correlated DEGs in the TCGA-HNSCC dataset. WikiPathway analyses showing the terms enriched for the top 1,500 DEGs correlated positively (D) and negatively (E) with p63.

**Figure S2.** Summary of the RNA-seq data from A253 and SCC25 cells. Pie charts showing the number of upregulated (red) and downregulated (blue) genes after the loss of p63 expression in A253 (A) and SCC25 (B) cells. GO terms enriched for the upregulated genes after p63 depletion in A253 (C) and SCC25 (D) cells. GO terms enriched for the downregulated genes after p63 depletion in A253 (E) and SCC25 (F) cells. Go term enrichment was performed using Metascape

**Figure S3.** Summary of p63 CHIP-seq data. (A) Scatter plots showing the correlation between biological CHIP-seq replicates in A253 and SCC25 cells. (B) Motif analysis showing the top motifs by occurrence in the ChIP-seq studies. (C) Pie charts showing the distribution of p63 binding sites across A253 and SCC25 genomes. (D) Profile plots showing the dynamics of poised enhancer (H3K4me1), active enhancer (H3K27ac), and promoter (H3K4me3) marks around p63 binding sites in A253 and SCC25 cells. p63 binding sites were clustered into three groups by k-means clustering. The average histone signal is depicted in black. Dark colors represent the limits of 50% of the signal, whereas light colors represent the limits of 90% of the signal. (E) Biological processes enriched for genes located within the regulatory regions represented in each cluster.

**Figure S4.** Transcriptomic changes accompanying the loss of p63 in SCC25 cells. (A) Western blot showing the robust shRNA-mediated depletion of p63 in SCC25 cells. (B) Genomic maps showing the super enhancer regions along the genomic locus of FST on chromosome 5 in both A253 and SCC25 cells.

**Figure S5.** Heat map showing the expression of genes in the TGF-β superfamily in HNSCC. (Top) Expression of genes for ligands that activate SMAD signaling pathways, sorted by decreasing expression in cancer tissues. TGFB1, INHBA (activin A), and BMP7 are the most highly expressed ligands in HNSCC. (Bottom) Expression of genes for TGF-β receptors, sorted by decreasing expression in cancer. TGFBR2 and ACVR1 (activin receptor) are the top receptors expressed. (Bottom) FST is the second-highest-expressed inhibitor of this pathway in HNSCC.

**Figure S6.** Loss of FST blocks proliferation and promotes invasion. (A) Bar graph showing the loss of proliferation in A253 cells following the loss of FST. (B) Bar graph showing the loss of proliferation in SCC cells following the loss of FST. (C) Bar graph showing the increase in invasion in A253 cells following the loss of FST. Representative images are shown in left panel. (D) Bar graph showing the increase in invasion in SCC25 cells following the loss of FST. Representative images are shown in left panel. Data presented as mean±standard deviation SD (n≥3). Statistical significance was determined using unpaired Student’s *t* tests: ***P < 0.001, ****P < 0.0001.

**Figure S7.** p63 mediates EGF-driven FST expression in CAL27 cells. (A) Western blot showing the increase of FST protein levels after EGF stimulation. (B) Western blot showing the absence of an EGF-mediated increase of FST levels in p63-depleted CAL27 cells despite robust activation of ERK1/2.

**Figure S8.** CAL27 FST models. Western blots showing the successful generation of FST-depleted (A) and FST-overexpressing (B) CAL27 cells.

**Figure S9.** UMAP plots showing the expression of the various genes used to annotate the single-cell RNA-seq data set reported by Puram *et al.* ([44](#_ENREF_44)).

**Table S1:** Data from the combined TCGA and Cell-line Analysis. (Tab 1) Correlation scores of genes in comparison to p63 expression in the TCGA dataset. (Tab 2) Results from the differential gene expression analysis comparing control to normal tissues from the TCGA dataset. (Tab 3) The 11,790 DEGs with foldchange values matching predicted correlation scores. (Tab 4) The 430 p63 correlated genes obtained from the combined analysis of the TCGA and cell line datasets.

**Table S2:** Summary of ChIP-sequencing data. (Tab 1) The 13,800 p63 ChIP-seq identified by irreproducible discovery rate (IDR) analysis in A253 cells. (Tab 2) The 37,516 p63 ChIP-seq sites identified by IDR analysis in SCC25 cells.
