# Supplementary figures and images for: An integrated genomic approach identifies follistatin as a target of the p63-epidermal growth factor receptor oncogenic network in head and neck squamous cell carcinoma
Source: NAR Cancer. 2023 Jul 24;5(3):zcad038. doi: 10.1093/narcan/zcad038 (PMC10365026; doi:10.1093/narcan/zcad038)

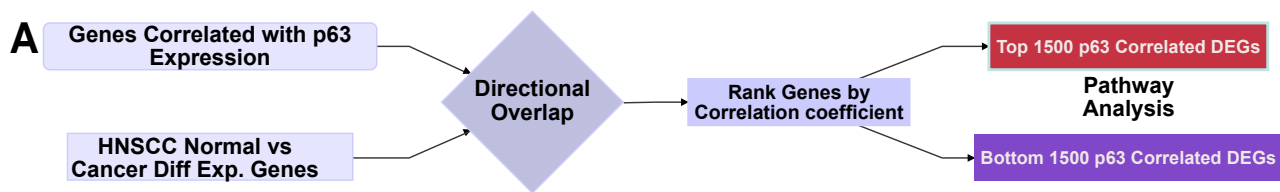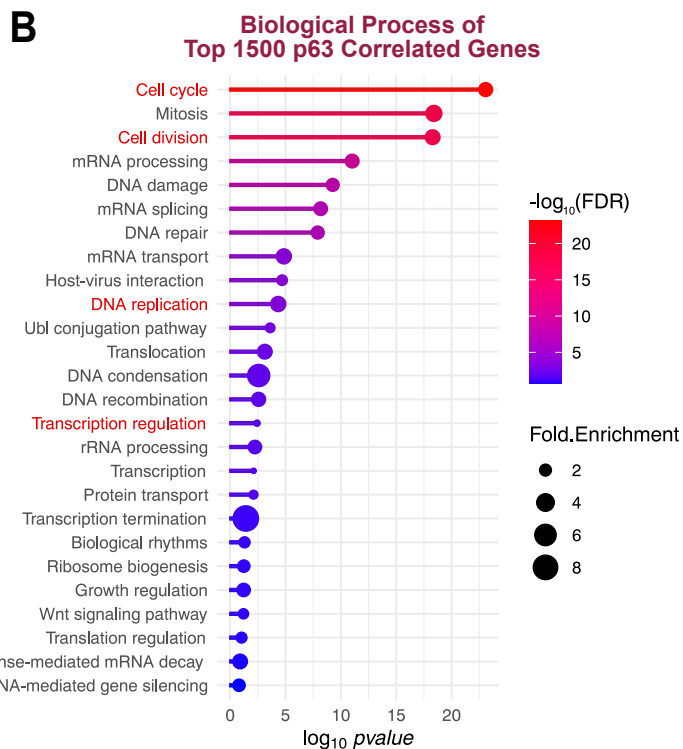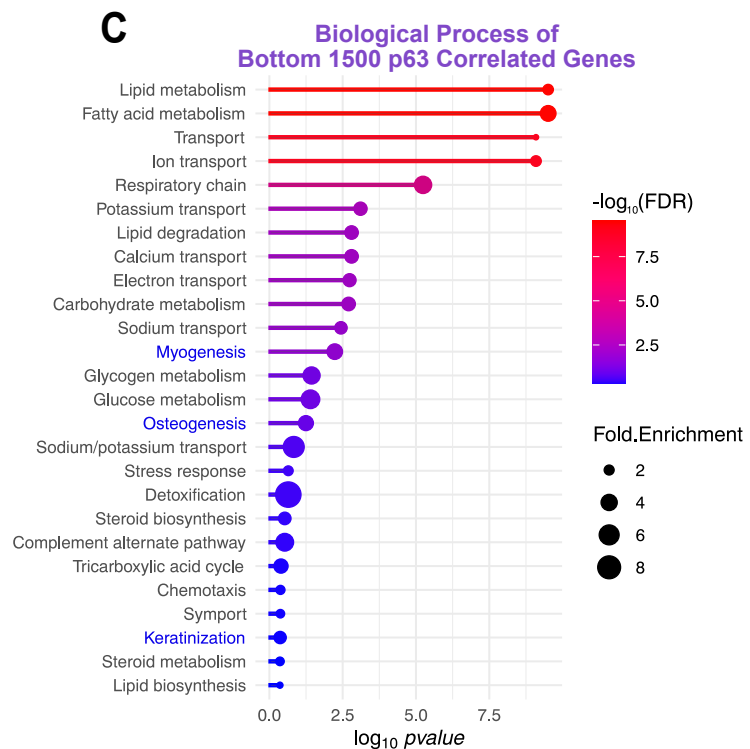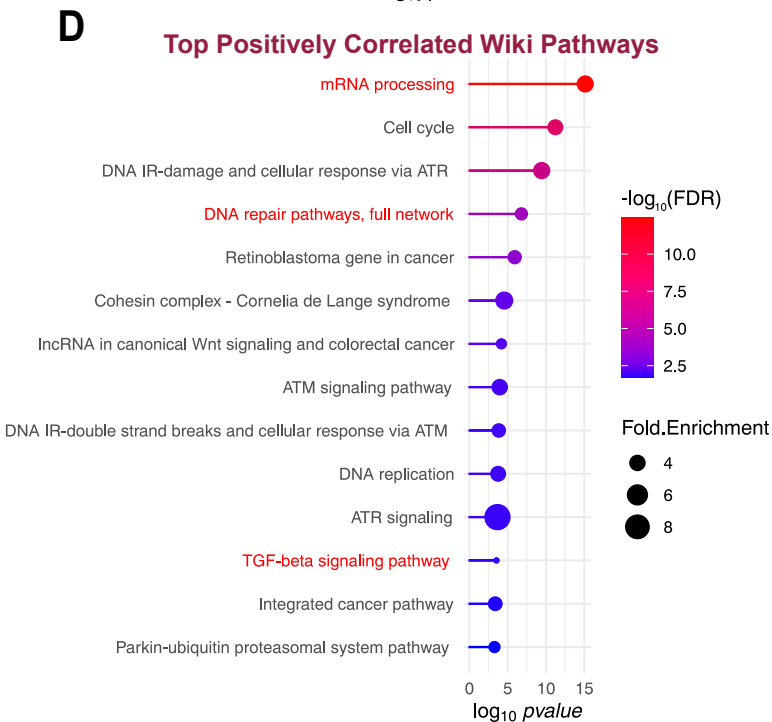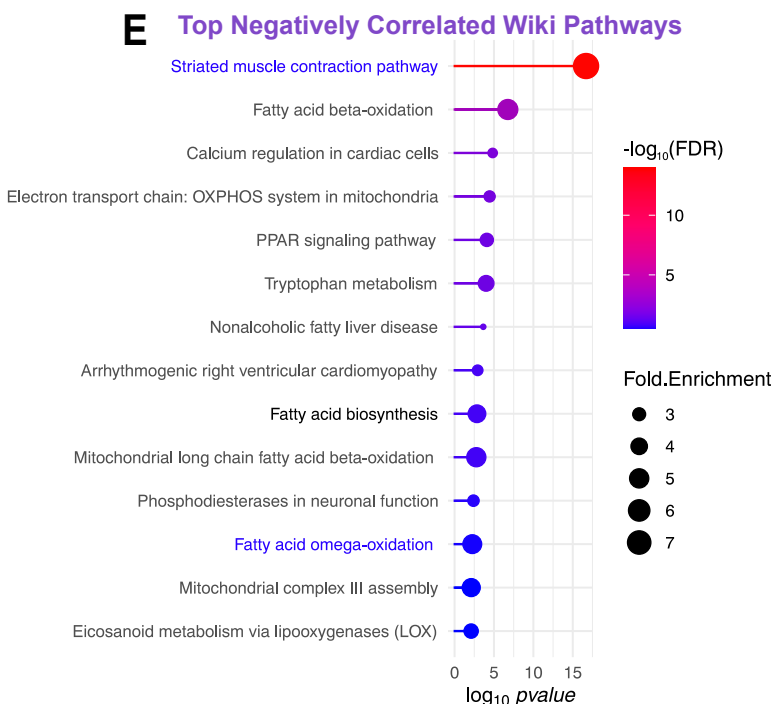

**Figure S1**

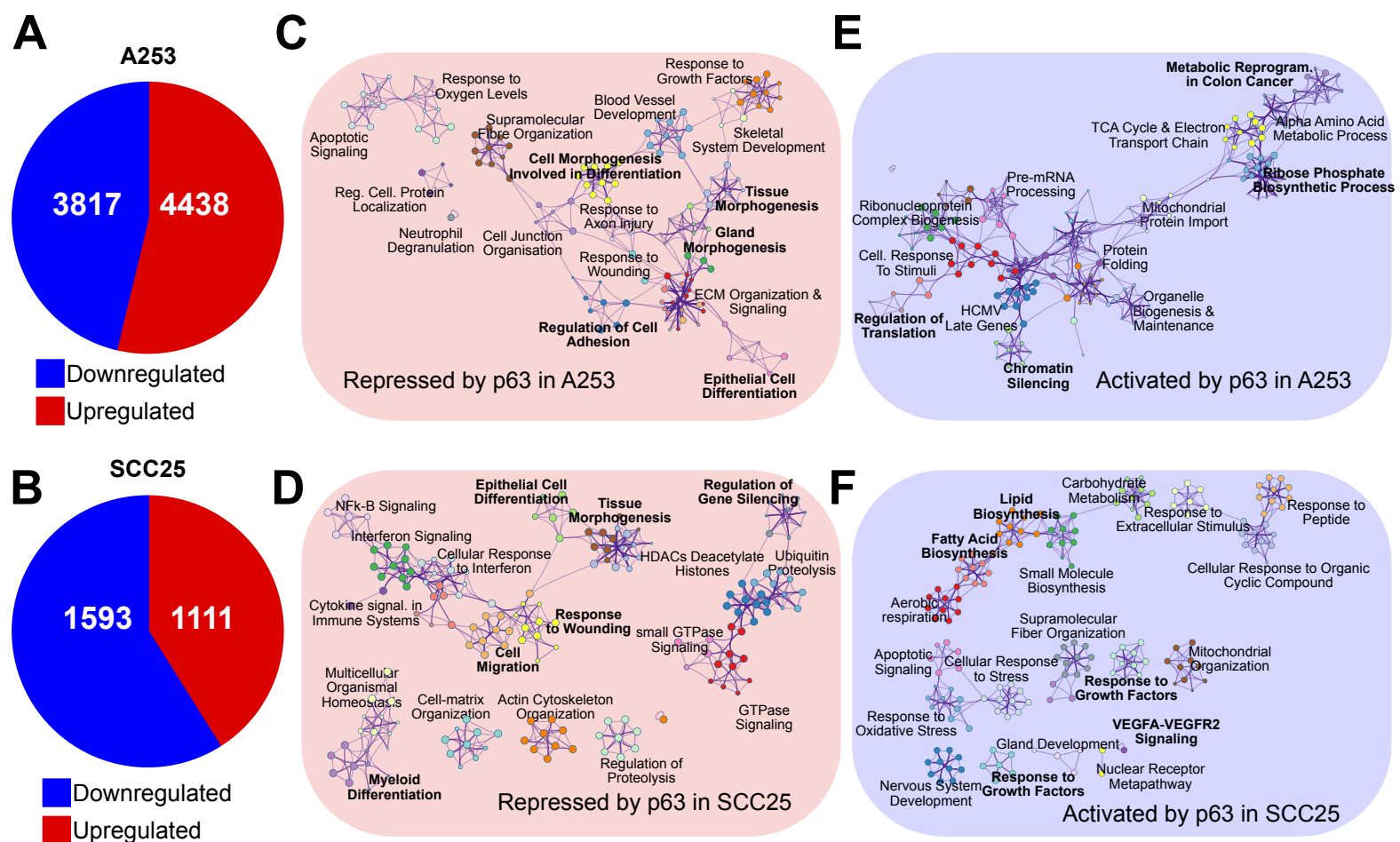

**Figure S2**

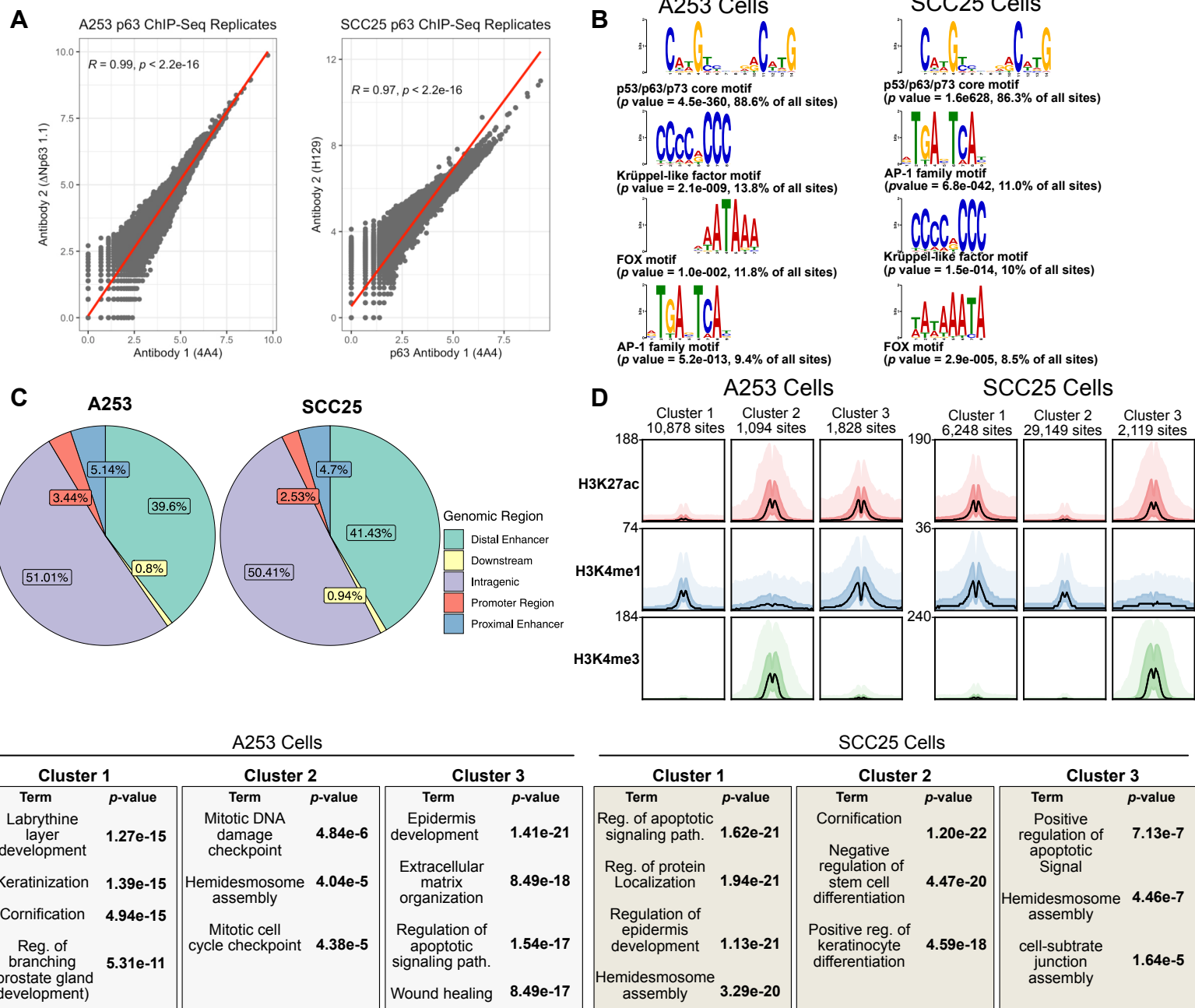

Figure S3

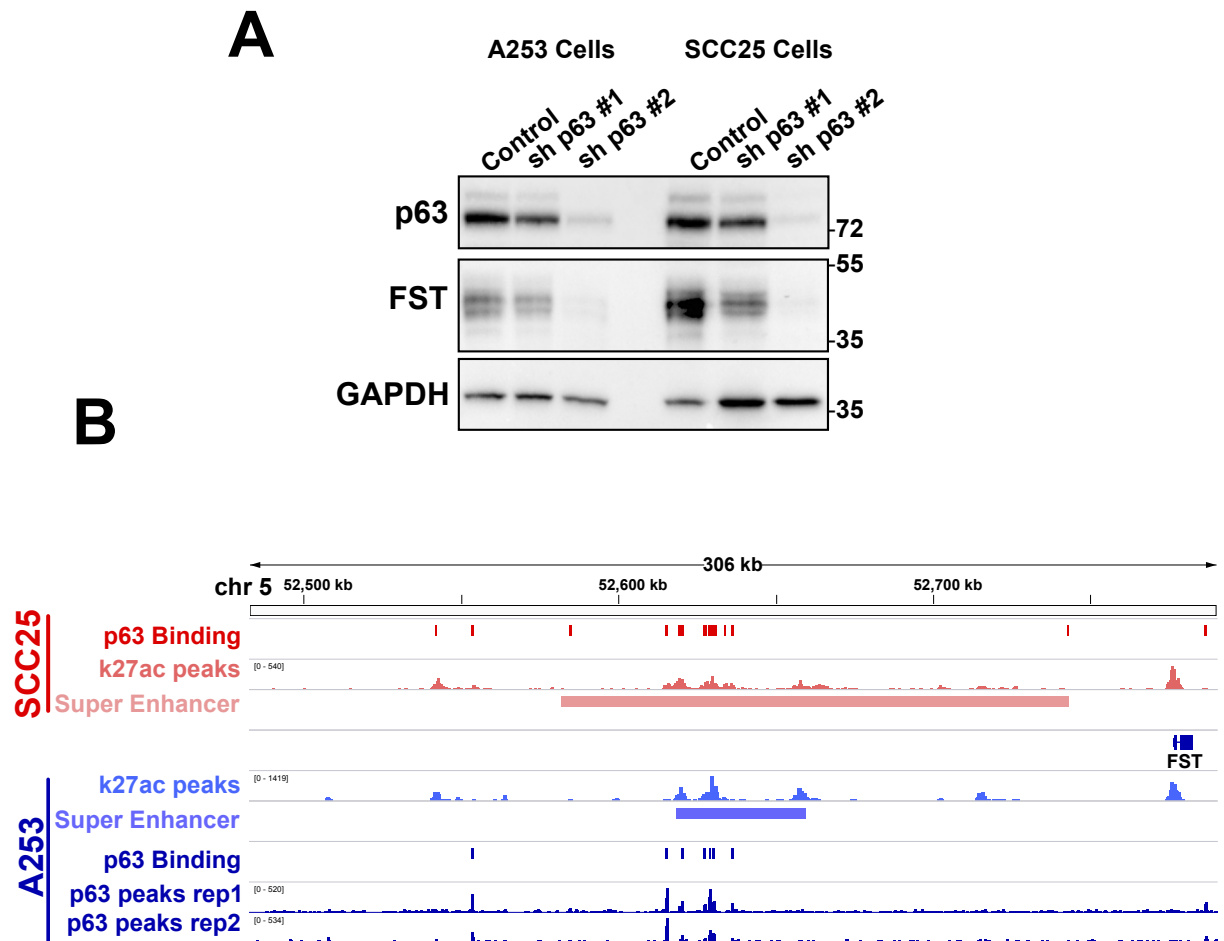

**Figure S4**

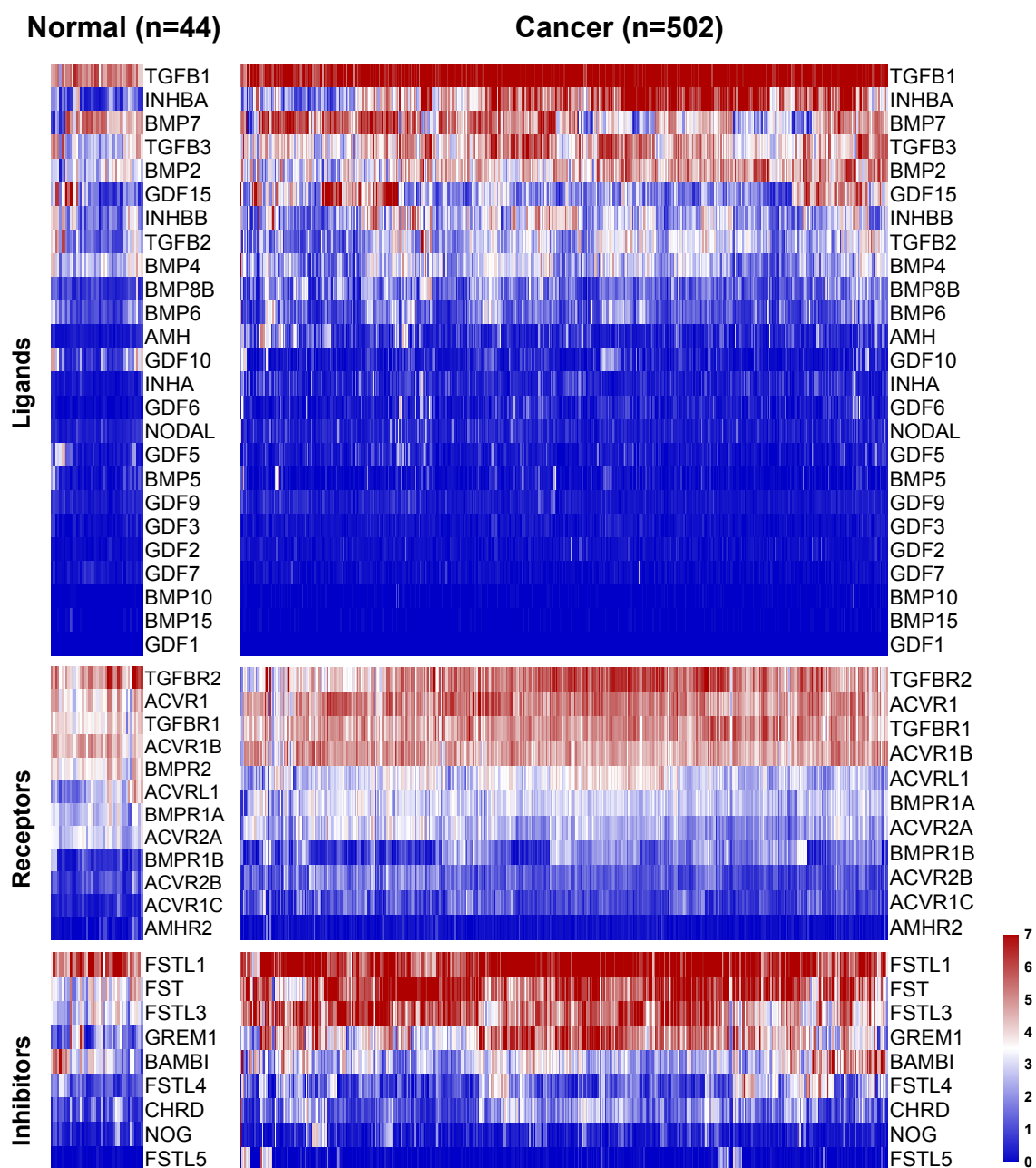

**Figure S5**

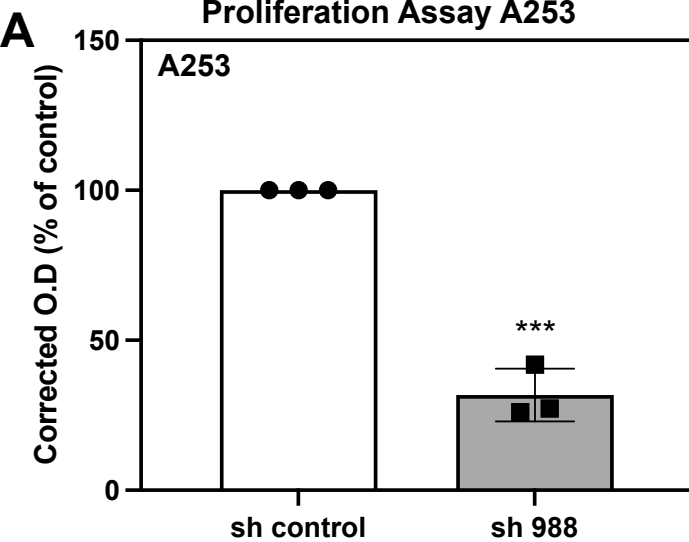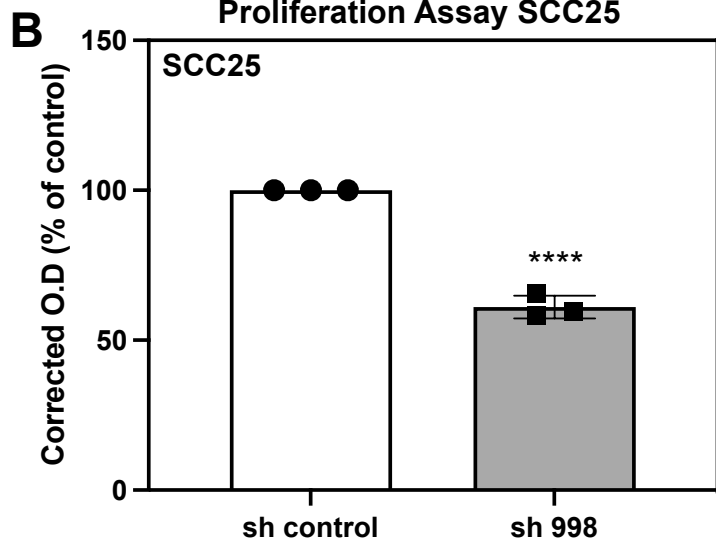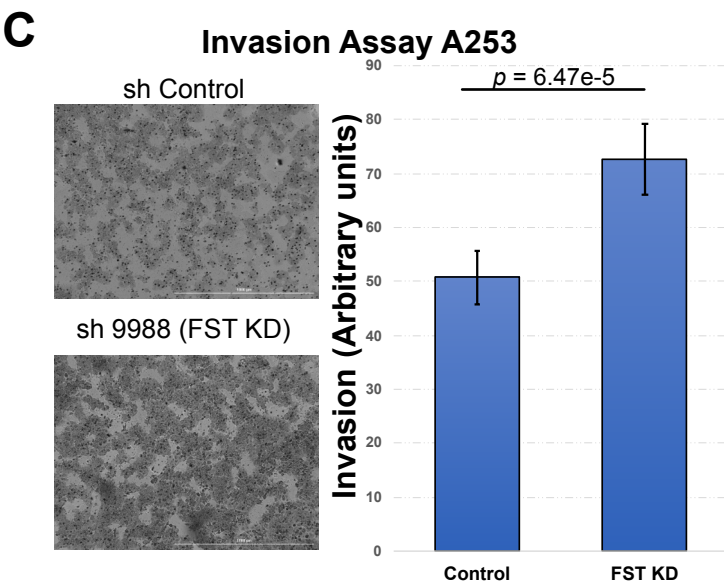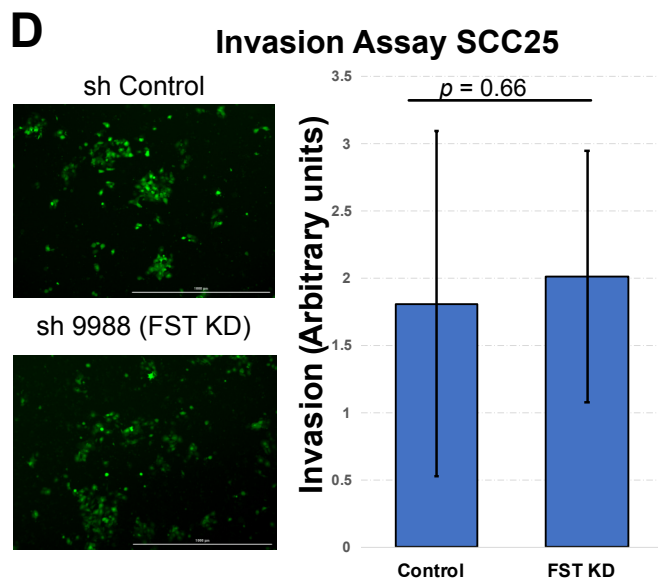

**Figure S6**

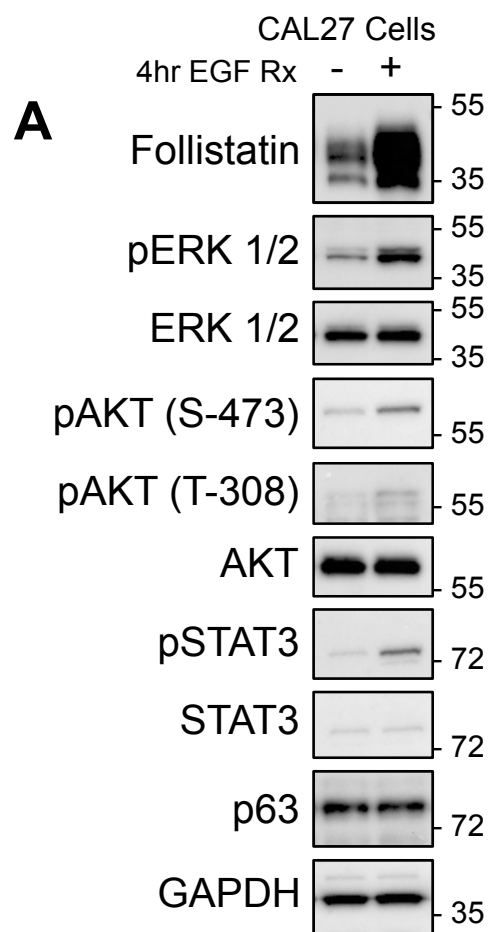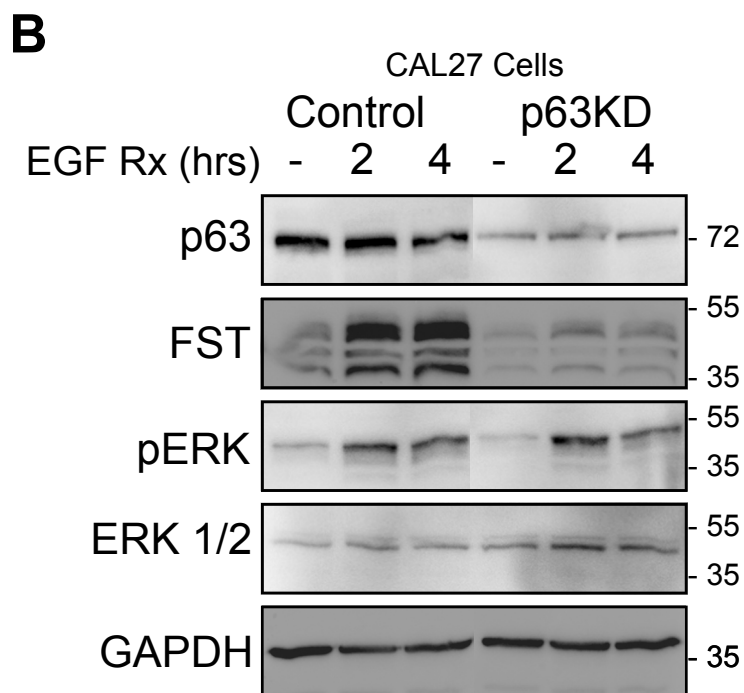

**Figure S7**

**CAL27**

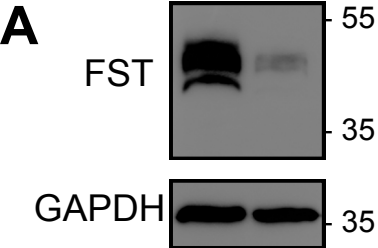

**CAL27**

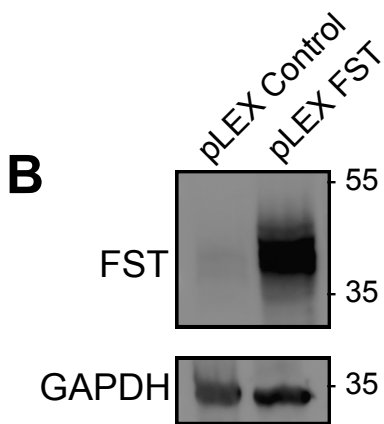

**Figure S8**

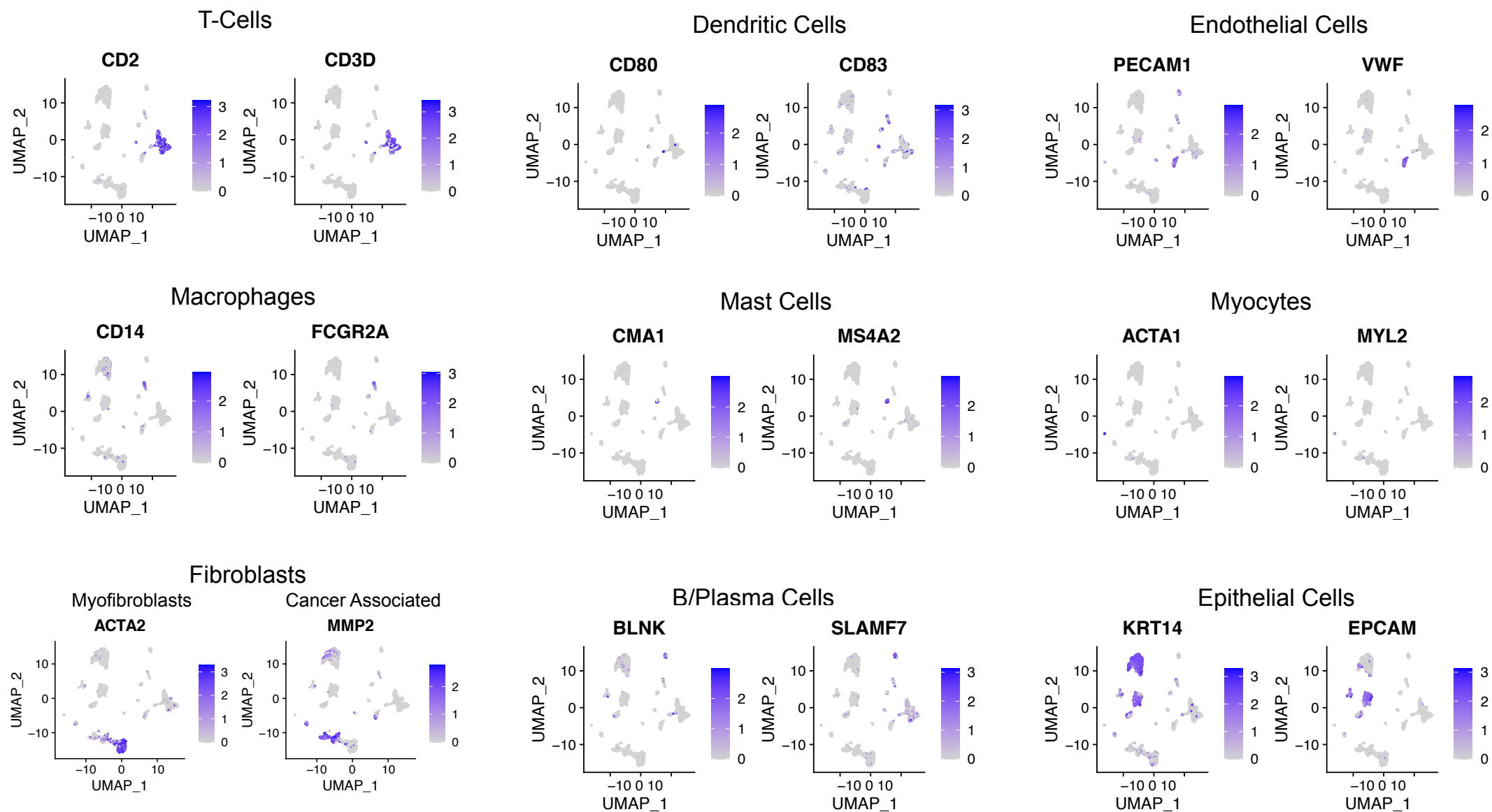

**Figure S9**

Supplement: zcad038_Supplemental_Files [file zcad038_supplemental_files.zip › Supplemental Figures Revision.pdf]
